# Generative AI for Personalized Learning Platforms in Otolaryngology–Head and Neck Surgery Education: Scoping Review

**DOI:** 10.2196/91785

**Published:** 2026-10-19

**Authors:** Jiang-Tao Zhong, Zai-Zai Cao, Shui-Hong Zhou

**Affiliations:** 1 Department of Otolaryngology, School of Medicine, First Affiliated Hospital Zhejiang University, Qingchun Road 79, Hangzhou, Zhejiang, 310003, China, 86 13868060120

**Keywords:** generative AI, large language models, ChatGPT, personalized learning, otolaryngology–head and neck surgery, otolaryngology education, ENT education, medical education, scoping review

## Abstract

**Background:**

Generative AI, including large language models (LLMs), may enable personalized learning in medical education. In otolaryngology–head and neck surgery (ear, nose, and throat [ENT]) education, AI may support knowledge retrieval, adaptive assessment, feedback, and scalable learning, but evidence remains fragmented.

**Objective:**

This scoping review aimed to map generative AI research in otolaryngology education; summarize applications, performance, benefits, and risks; and identify directions for AI-enabled personalized learning.

**Methods:**

We included English-language empirical studies, preprints, and technical or developmental evaluations published from 2017 through 2026 with direct relevance to professional ENT education; nonempirical publications and studies without a professional educational focus were excluded. PubMed, Embase, Web of Science, Scopus, ERIC, IEEE Xplore, and CINAHL were searched, supplemented by citation searching. The final search was conducted on July 6, 2026. Data were charted using a standardized form by 2 reviewers and synthesized through a 3-stage inductive thematic analysis, with third reviewer adjudication. No formal critical appraisal or meta-analysis was performed because of study heterogeneity.

**Results:**

A total of 42 studies published between 2023 and 2026 were included. Most were performance studies (n=33, 78.6%), followed by system development (n=3, 7.1%), educational intervention studies (n=3, 7.1%), surveys (n=2, 4.8%), and guideline development (n=1, 2.4%). Most studies evaluated question sets or model outputs rather than participants. Applications included knowledge retrieval, revision, board-style question practice, adaptive assessment, AI avatar–delivered instruction, and operative-planning support. Seven major thematic domains were identified: (1) educational support and learning enhancement; (2) learning outcomes, educational effectiveness, and pedagogical value; (3) model performance, accuracy, and variability; (4) assessment, examination readiness, and question-type sensitivity; (5) reliability, safety, and risks; (6) adoption, usability, and implementation barriers; and (7) advanced AI architectures and future educational design. Newer models performed strongly on selected structured knowledge tasks, but performance varied by task, difficulty, modality, and reasoning demands. Evidence remained heterogeneous and benchmarking focused, with only 3 educational interventions and limited evidence for sustained learning, clinical reasoning, or real-world outcomes. Hallucinations, unreliable sourcing, confidently incorrect outputs, and contextual and multimodal reasoning limitations supported supervised rather than autonomous use.

**Conclusions:**

By moving beyond reviews centered on AI applications or model performance, this review provides an education-focused synthesis linking generative AI capabilities, learning outcomes, assessment, safety, implementation, and emerging architectures to the design of personalized learning in otolaryngology. Its contribution is a conceptual shift from evaluating standalone chatbots toward developing supervised, pedagogically governed, knowledge-grounded, and adaptive learning systems that preserve learner reasoning and expert oversight. These findings inform educators, curriculum designers, and developers when integrating generative AI into formative assessment, feedback, remediation, and personalized learning activities, while avoiding unsupported autonomous or high-stakes use. Future research should prioritize longitudinal educational outcomes, curriculum integration, higher-order reasoning, equitable implementation, and governance.

## Introduction

### Rationale

Otolaryngology–head and neck surgery (ear, nose, and throat [ENT]) is a broad and technically demanding specialty that requires trainees to integrate complex knowledge across anatomy, oncology, airway management, otology, rhinology, pediatrics, and perioperative care. However, in many training systems, undergraduate and early postgraduate exposure to ENT remains limited, and protected teaching time is constrained by clinical service demands. These structural constraints have intensified the need for scalable, flexible, and learner-centered educational approaches that support self-directed learning and just-in-time knowledge acquisition [1].

Medical education is undergoing a rapid digital transformation, with increasing emphasis on personalization, adaptive learning pathways, and competency-based progression. Generative AI, particularly large language models (LLMs), has emerged as a general-purpose technology relevant to these educational goals. Systems such as ChatGPT, Gemini, Claude, and related multimodal models are capable of generating explanations, synthesizing information, answering questions, and simulating clinical scenarios in natural language, with broad applications across biomedical knowledge domains [2,3]. More broadly, AI has been increasingly discussed as an enabling component of digitally supported, person-centered learning and care pathways in medicine [4].

Within otolaryngology, interest in generative AI has grown rapidly. The specialty has been identified as a suitable testbed for AI-assisted educational and clinical applications due to its reliance on imaging, structured anatomical knowledge, guideline-based decision pathways, and subspecialty-diverse training requirements [5,6]. Broader surgical education literature has also emphasized the potential role of AI in supporting training, assessment, and simulation [7]. At the same time, the clinical AI literature highlights that generative AI differs fundamentally from traditional educational resources and clinical decision-support systems. Its outputs are probabilistic, model dependent, and sensitive to task framing, raising questions regarding transparency, accountability, and governance in medical contexts [8-10]. These considerations apply equally to educational uses and underscore the need for systematic, domain-specific evaluation rather than premature adoption.

Several published reviews and perspective articles have examined AI and generative AI in otolaryngology, but their scope differs from the educational focus of this review. Lechien [11] reviewed generative AI across otolaryngology, with emphasis on the accuracy and clinical utility of ChatGPT for disease information, clinical vignettes, and scientific information. Edalati et al [12] examined opportunities, technical limitations, and ethical considerations of GPT-4 in otolaryngology, with education considered alongside diagnosis, perioperative care, research, and administrative applications. Crowson and Nwosu [13] addressed integration of AI into otolaryngology practice, particularly data infrastructure, electronic health records, model validation, and clinical implementation. From an educational perspective, Brennan et al [1] described ways in which ChatGPT could supplement ENT surgical training, but their work was a trainee-centered perspective based on illustrative prompts rather than a systematic synthesis of evidence. Buess et al [14] conducted a scoping review of generative and multimodal AI across medicine, focusing primarily on model architectures, datasets, clinical applications, and evaluation methods rather than specialty-specific education.

Thus, existing reviews have primarily addressed the clinical and technical applications of AI in otolaryngology or the broader evolution of generative AI across medicine [15-17]. They have not systematically mapped the empirical literature specifically on generative AI in ENT professional education or integrated evidence across educational applications, learning outcomes, model performance, assessment, safety, adoption, and emerging system architectures for personalized learning. This distinction is important because strong performance on isolated knowledge or examination tasks does not establish educational effectiveness, curricular value, or readiness for implementation. A dedicated scoping review is therefore needed to characterize this heterogeneous and rapidly evolving evidence base, identify evidence gaps, and determine how current findings can inform pedagogically grounded, AI-enabled personalized learning platforms in otolaryngology.

### Objectives

The aim of this scoping review was to systematically map literature on generative AI in otolaryngology education. Specifically, we sought to characterize educational contexts and applications studied; summarize how generative AI has been evaluated in this field; and identify key conceptual, methodological, and practical gaps. We also aimed to synthesize the emerging directions of research and development to inform pedagogically grounded, AI-enabled personalized learning platforms in otolaryngology education and provide a conceptual foundation for future empirical and implementation research. The review was conceptualized around generative AI–enabled personalized learning as the central concept; professional otolaryngology education as the context; and learners, educators, assessment tasks, and educational systems as the relevant units of inquiry. The guiding research question for this review was as follows: “What is the current state of evidence regarding the use of generative AI for personalized learning in otolaryngology (ENT) education?” This question encompassed LLM performance, educational applications and implementation contexts, emerging platform-level designs, reported benefits and risks, and architectural strategies for adaptive and personalized learning.

## Methods

### Protocol and Registration

This scoping review was conducted and reported in accordance with the PRISMA-ScR (Preferred Reporting Items for Systematic Reviews and Meta-Analyses extension for Scoping Reviews) checklist and the methodology described by Tricco et al [18]. A review protocol was developed before the review and is provided in Multimedia Appendix 1. The protocol was not prospectively registered.

### Eligibility Criteria

Eligible sources were English-language original empirical studies, preprints, and technical or developmental evaluations with direct relevance to professional otolaryngology education. Studies were eligible if they directly evaluated or applied generative AI in ENT professional education and reported empirical data, performance evaluations, user studies, educational interventions, or developmental outcomes. Review articles, commentaries, expert perspectives, letters, conference abstracts, purely theoretical or technical papers without an educational component, and studies focused on patient education rather than professional training were excluded. Publications from 2017 through 2026 were considered to capture the modern era of generative AI relevant to medical education [19].

### Information Sources

We searched PubMed (NCBI [National Center for Biotechnology Information]), Embase (Elsevier), Web of Science (Clarivate), Scopus (Elsevier), ERIC, IEEE Xplore, and CINAHL (EBSCOhost). Each database was searched separately; no multidatabase platform was used simultaneously. Citation searching was also used to identify additional eligible sources. Specifically, included studies underwent backward and forward citation searches. No study registries or additional online or print sources were searched or browsed. No authors, experts, manufacturers, or other parties were contacted, and no other information sources or search methods were used. The final search was conducted on July 6, 2026.

### Search

The literature search was reported in accordance with the PRISMA-S (Preferred Reporting Items for Systematic Reviews and Meta-Analyses literature search extension) guideline [20]. The search strategy combined controlled vocabulary terms and free-text keywords related to otolaryngology, head and neck surgery, AI, generative AI, LLMs, ChatGPT, DeepSeek, education, training, personalized learning, teaching, and assessment. Search limits included the English language and publication years from 2017 to 2026. No published search filters were used, and no search strategies from previous literature reviews were adapted or reused. No search update was performed after July 6, 2026. No formal peer review of the search strategy was conducted. Complete search strategies for all 7 databases, reproduced exactly as run, are provided in Multimedia Appendix 2.

### Selection of Sources of Evidence

All retrieved records were imported into EndNote (version 20; Clarivate) and deduplicated using its automated duplicate-detection function before screening. Two reviewers independently screened titles and abstracts and assessed potentially eligible full-text reports against the predefined eligibility criteria. Disagreements were resolved through discussion and, when necessary, adjudication by a third reviewer. Citation searching was used to identify additional potentially eligible reports.

### Data Charting Process

Data were charted using a standardized form developed iteratively by the review team. Two reviewers independently charted data, and discrepancies were resolved through discussion or adjudication by a third reviewer.

### Data Items

For each included study, we recorded publication year, country, study type, purpose, setting and context, generative AI system evaluated or used, sample size or evaluation set, and findings relevant to the thematic synthesis. Study types were assigned using each article’s title and the design information reported in its Abstract and Methods sections. Explicit author-reported design terms were used when available; otherwise, the primary objective and methodological approach determined the category. Studies were mapped to 5 standardized categories: performance study, survey study, system development study, educational intervention study, and guideline development study.

### Critical Appraisal of Individual Sources of Evidence

No formal critical appraisal of individual sources of evidence was conducted because this scoping review aimed to map a heterogeneous emerging literature rather than estimate intervention effectiveness or pooled effects.

### Synthesis of Results

The charted data were synthesized descriptively and through a 3-stage inductive thematic synthesis. First, JTZ and ZZC independently summarized the subthemes identified in each included article. Second, SHZ deduplicated, screened, and refined the 2 sets of subthemes. Third, JTZ and ZZC synthesized the refined subthemes into major themes; SHZ adjudicated disagreements. The final synthesis comprised 7 major themes and associated subthemes. Results were summarized narratively, from source characteristics to thematic synthesis across the 7 domains.

## Results

### Selection of Sources of Evidence

The final database search was conducted on July 6, 2026. The 7 databases yielded 10,757 records: PubMed (n=4786, 44.5%), Embase (n=4474, 41.6%), Web of Science (n=538, 5.0%), Scopus (n=605, 5.6%), ERIC (n=7, 0.1%), IEEE Xplore (n=44, 0.4%), and CINAHL (n=303, 2.8%). Before screening, duplicate records (n=1918, 17.8%), records published before 2017 (n=761, 7.1%), and 1 non-English record (n=1, <0.1%) were removed. The remaining 8077 (75.1%) database records were screened by title and abstract, and 7897 (73.4%) records were excluded as not relevant to generative AI in otolaryngology education. The full texts of 180 (1.7%) database records were sought and assessed for eligibility. One additional report identified through citation searching (backward and forward citation searches) was also retrieved and assessed; no reports were unavailable for retrieval.

Of the 180 database reports assessed at full text, 139 (77.2%) articles were excluded for the following reasons: not focusing on generative AI (n=12, 6.7%), not focusing on ENT (n=10, 5.6%), not focusing on medical education (n=50, 27.8%), focusing primarily on patient education rather than professional training (n=50, 27.8%), or being reviews, commentaries, expert perspectives, letters, or conference abstracts (n=17, 9.4%). No reports were excluded due to retrieval failure. In total, 41 (22.8%) database studies and 1 (0.6%) citation-identified study were included, yielding 42 (23.3%) studies, including 1 (0.6%) preprint. The study selection process is summarized in the PRISMA flow diagram (Figure 1).

**Figure 1.**
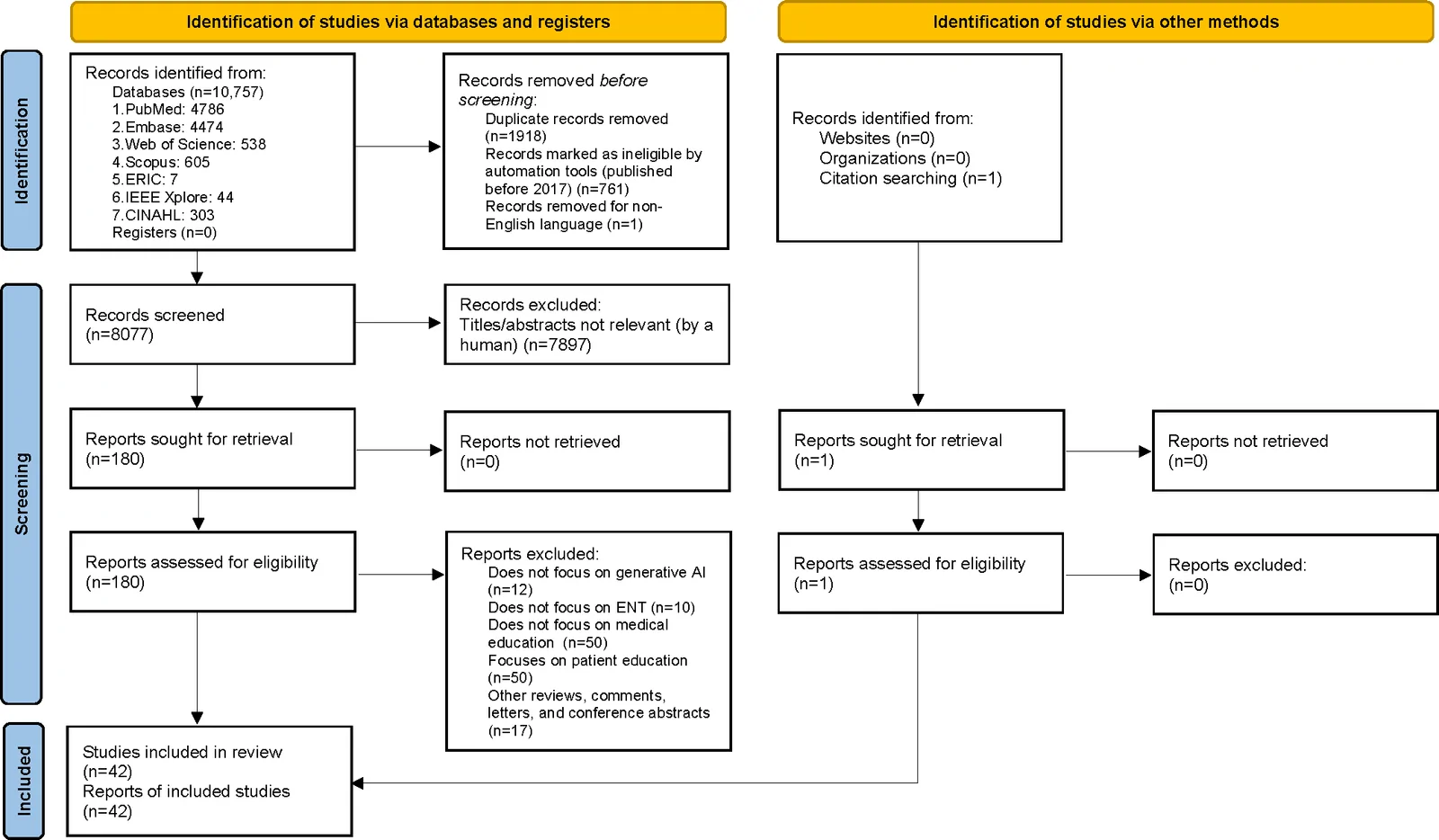
PRISMA-ScR (Preferred Reporting Items for Systematic Reviews and Meta-Analyses extension for Scoping Reviews) flow diagram of search and screening for large language models in otolaryngology education. ENT: ear, nose, and throat.

### Characteristics of Sources of Evidence

The 42 included studies were published between 2023 and 2026: 7 (16.7%) in 2023, 17 (40.5%) in 2024, 13 (31%) in 2025, and 5 (11.9%) in 2026. The evidence base was internationally distributed across North America, Europe, and Asia, including several multinational collaborations. Most studies were performance studies (33/42, 78.6%), followed by 3 (7.1%) system development studies, 3 (7.1%) educational intervention studies, 2 (4.8%) survey studies, and 1 (2.4%) guideline development study. Educational contexts were predominantly assessment-focused and benchmarking-focused, especially board-style or standardized question banks, while a smaller group addressed adaptive knowledge learning, resident induction, operative-planning support, study-guide generation, trainee adoption, and specialized AI system development. Table S1 in Multimedia Appendix 3 provides the study-level details, and Figures 2–5 provide the review-level evidence mapping. The evaluated technologies ranged from general-purpose generative AI models to customized or hybrid educational systems, including retrieval-augmented, agentic, adaptive-assessment, and AI avatar–based approaches (Table 1).

**Figure 2.**
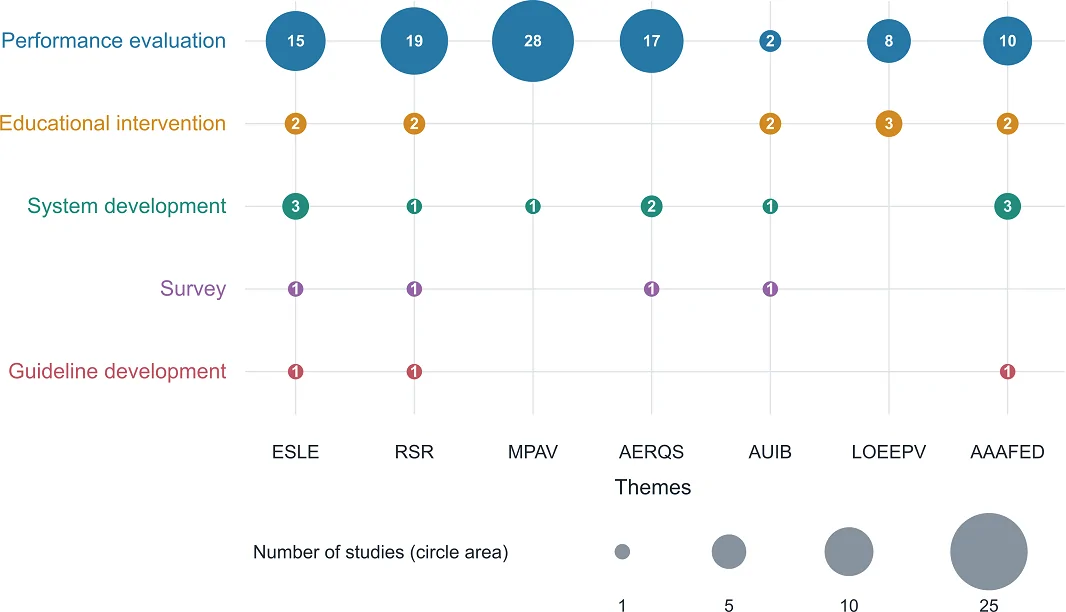
Evidence map of study types and themes in generative AI research in otolaryngology education. Circle area is proportional to the number of studies, with exact counts shown within circles. Colors indicate study types, and empty cells represent zero studies. Individual studies may address multiple themes. AAAFED: advanced AI architectures and future educational design; AERQS: assessment, examination readiness, and question-type sensitivity; AUIB: adoption, usability, and implementation barriers; ESLE: educational support and learning enhancement; LOEEPV: learning outcomes, educational effectiveness, and pedagogical value; MPAV: model performance, accuracy, and variability; RSR: reliability, safety, and risks.

**Figure 3.**
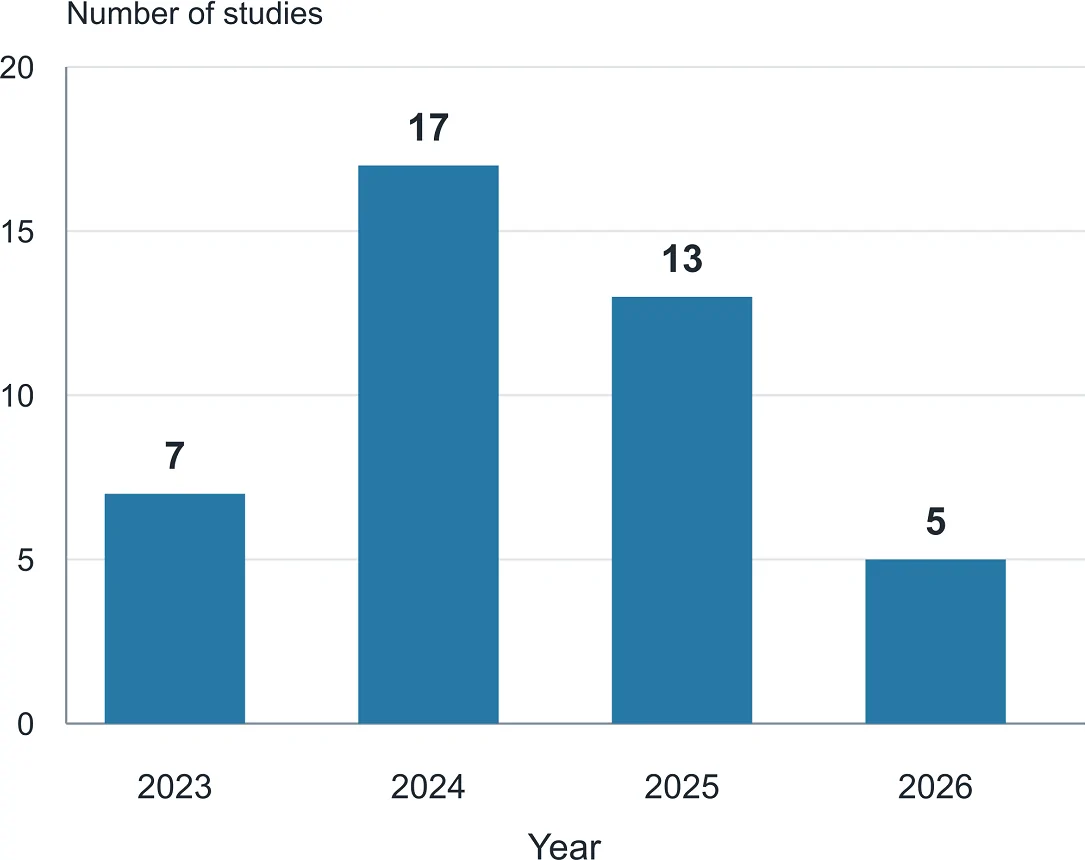
Publication-year distribution of included studies. Bars show the number of included studies published in each year from 2023 to 2026 (N=42). Numbers above the bars indicate exact counts.

**Figure 4.**
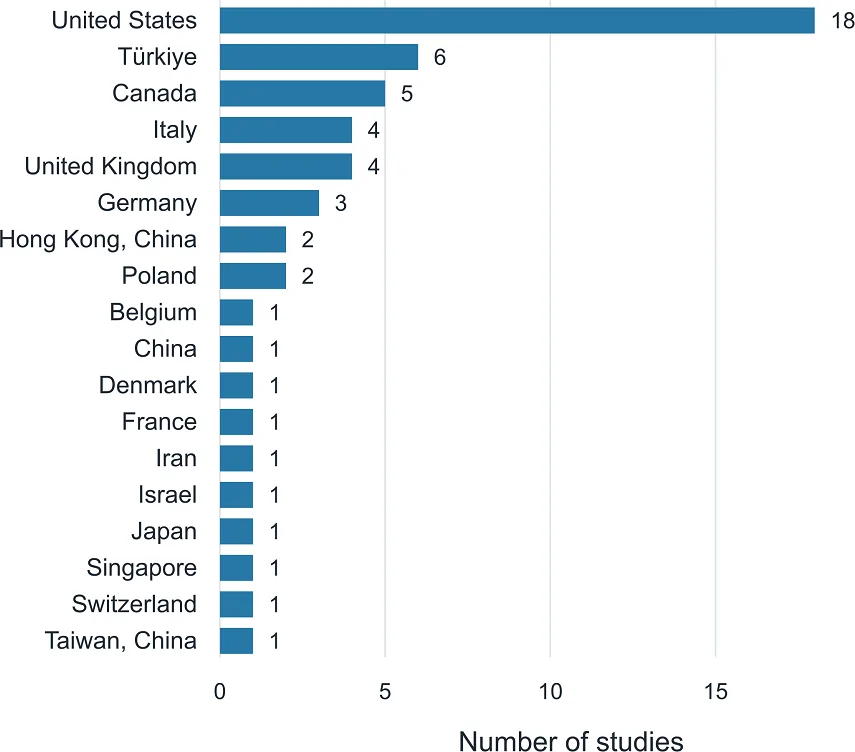
Number of included studies by country/region. Horizontal bars show study counts for each country/region, with exact counts displayed at the end of each bar. Multicountry studies were counted once for each participating country/region, yielding 54 study-location contributions across 42 studies.

**Figure 5.**
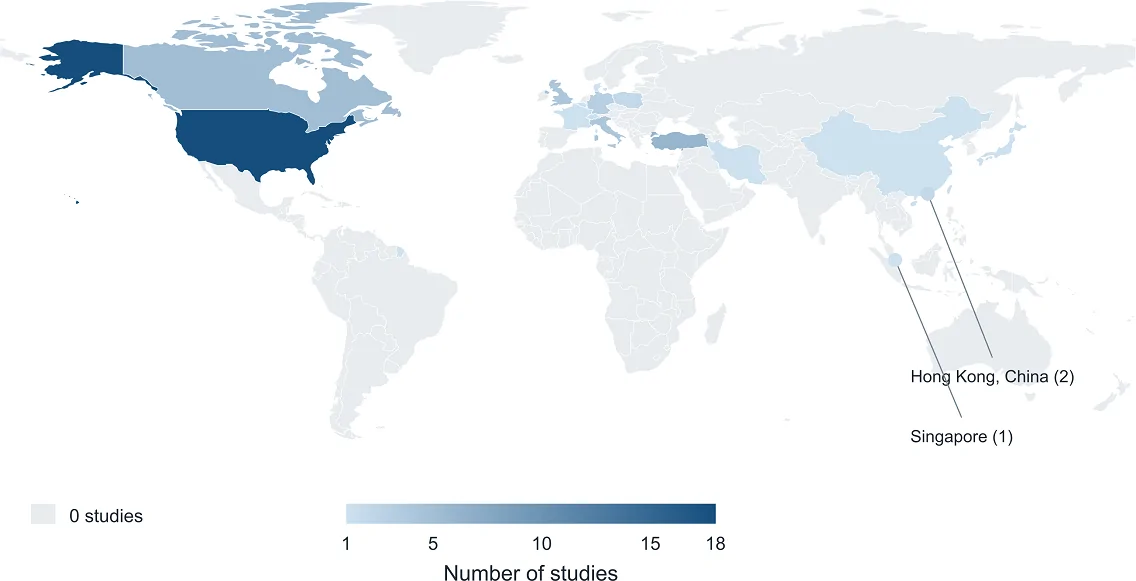
Global distribution of included studies. Color intensity indicates the number of studies associated with each country/region; darker blue represents higher counts, and gray indicates zero included studies. Labeled markers identify Hong Kong, China, and Singapore to improve visibility. Multicountry studies were counted once for each participating country/region, yielding 54 study-location contributions across 42 studies.

**Table 1.** Large language models reported in the identified literature.

Model	Interface	Developer	Year of release
GPT-3.5	ChatGPT	OpenAI	2022
GPT-3.5 Turbo	ChatGPT	OpenAI	2023
GPT-4	ChatGPT	OpenAI	2023
GPT-4 Turbo	ChatGPT	OpenAI	2023
GPT-4 with Vision (GPT-4V)	ChatGPT	OpenAI	2023
Custom GPT-4 ENT^a^ Expert MCQ^b^ Solver	OpenAI custom GPT builder interface	OpenAI	2023
E-GPT-A (custom GPT-4)	OpenAI custom GPT builder interface	OpenAI	2023
GPT-4o	ChatGPT	OpenAI	2024
GPT-4o mini	ChatGPT	OpenAI	2024
ChatENT (GPT-4 plus CEKB^c^/RAG^d^)	Custom virtual Q&A^e^ platform	OpenAI base model; developed by study authors	2024
ChatGPT-5	ChatGPT	OpenAI	2025
o3-mini	OpenAI API	OpenAI	2025
Google Bard	Bard	Google	2023
Gemini Ultra	Gemini	Google	2023
Gemini 1.0 Pro	Gemini	Google	2023
Gemini 1.5 Flash	Gemini	Google	2024
Gemini 1.5 Flash 8B	Gemini	Google	2024
Gemini 1.5 Pro	Gemini	Google	2024
Gemini 2.0	Gemini	Google	2024‐2025
Gemini-3	Gemini	Google	2025
Claude 2	Claude	Anthropic	2023
Claude 2.1	Claude	Anthropic	2023
Claude 3	Claude	Anthropic	2024
Claude 3 Haiku	Claude	Anthropic	2024
Claude 3.5 Sonnet	Claude	Anthropic	2024
Claude 3 Opus	Claude	Anthropic	2024
Claude 3.7 Sonnet	Claude	Anthropic	2024‐2025
Claude Sonnet 4	Claude	Anthropic	2024‐2025
Grok-2	Grok	xAI	2024
Grok-3	Grok	xAI	2025
Perplexity	Perplexity	Perplexity AI	2022
Copilot	Copilot	Microsoft	2023
DeepSeek-V3	DeepSeek	DeepSeek	2024
DeepSeek-R1	DeepSeek	DeepSeek	2025
DeepSeek-4	DeepSeek	DeepSeek	2025
OpenEvidence	OpenEvidence	OpenEvidence Inc	2023
Eli5a	Research assistant (explainability focused)	A multidisciplinary team	2025
MEKAS^f^	Web-based adaptive assessment system usable on personal computers and mobile devices	Research team at Cathay General Hospital and National University of Tainan	2024
Llama 3.1 70B	Agentic workflow implemented on LangGraph	Meta (Llama models)	2024
Llama-3-Groq-70B-Tool-Use	API- and tool-based execution (Google Search, PubMed, arXiv)	Groq (tool use model)	2024
Llama-4	Llama	Meta	2025
HeyGen AI video avatar system	Web-based, modular learning platform with prerecorded AI avatar–delivered video modules	HeyGen Inc	2023

a ENT: ear, nose, and throat.

b MCQ: multiple-choice question.

c CEKB: ChatENT’s knowledge base.

d RAG: retrieval-augmented generation.

e Q&A: question and answer.

f MEKAS: Multi-Expert Knowledge-Aggregated Adaptive Assessment Scheme.

### Critical Appraisal Within Sources of Evidence

No formal critical appraisal of the included sources of evidence was conducted; therefore, no critical appraisal results are reported.

### Results of Individual Sources of Evidence

Study-level data relevant to the review question are presented in Table S1 in Multimedia Appendix 3, including study design, purpose, educational context, generative AI system, sample or evaluation set, and thematic contribution. Detailed findings from individual sources that informed the thematic synthesis are presented in Table S2 in Multimedia Appendix 4 through source-linked subthemes and representative quotations.

### Synthesis of Results

#### Overview

Thematic synthesis of the 42 studies [21-62] identified 7 major domains and 213 associated subtheme entries, each linked to representative quotations in Table S2 in Multimedia Appendix 4. The domains were as follows: (1) educational support and learning enhancement; (2) learning outcomes, educational effectiveness, and pedagogical value; (3) model performance, accuracy, and variability; (4) assessment, examination readiness, and question-type sensitivity; (5) reliability, safety, and risks; (6) adoption, usability, and implementation barriers; and (7) advanced AI architectures and future educational design.

Overall, the evidence was dominated by benchmarking and model performance evaluations of knowledge retrieval, question answering, and examination performance, with comparatively limited research on learner-centered outcomes, longitudinal learning, educational interventions, and real-world implementation. The 7 themes broadly encompassed educational applications and outcomes, technical performance and reliability, and implementation and future system design.

#### Theme 1: Educational Support and Learning Enhancement

Across the included studies, generative AI was consistently portrayed as a flexible and scalable educational support resource that can supplement traditional ENT training by providing accessible, query-responsive support across different learning contexts and levels of training. Reported applications included facilitating professional discussion [27], bridging access to specialized knowledge [29], providing query-tailored explanations for early-stage learners [35], and supporting continuing medical education [46]. In this sense, AI is framed not as a replacement for educators but as a complementary educational tool alongside established learning resources and expert instruction [49,50].

A prominent dimension of educational support was the capacity of generative AI to retrieve, synthesize, and present knowledge efficiently. Advanced systems such as ENTAgents retrieved information from guidelines, textbooks, and academic literature within seconds, supporting efficient learning and revision [47]. ChatENT similarly streamlined access to specialty knowledge and reduced the need to manually search guidelines, textbooks, and academic papers [25]. In audiology, LLMs provided explicit reasoning processes that may help teachers and students rapidly identify key concepts and understand underlying reasoning processes [52]. Together, these findings suggest that the core educational contributions of generative AI lie in optimizing access to, organization of, and reasoning around knowledge rather than merely generating new content.

Beyond information access, several studies emphasized individualized learning support, formative feedback, self-assessment, and targeted remediation. LLMs such as GPT-4o provided immediate feedback on board-style questions and analyzed incorrect responses, helping learners identify specific knowledge gaps and pursue targeted remediation in a self-directed manner [48]. GPT-4 was proposed to generate personalized academic plans tailored to individual learning styles and abilities [29], while other models provided rapid feedback, explanations, and personalized remediation for examination preparation [61]. ChatGPT supported immediate self-assessment through past examination questions [60], while models with moderate-to-high board-question accuracy were proposed as study companions for basic knowledge reinforcement and practice-oriented learning [56]. These functions position generative AI as a mechanism for formative assessment, adaptive learning, self-assessment, and personalized remediation.

ChatGPT was reported to correctly interpret subtle domain-specific distinctions, including closely related anatomical concepts [27]. In pediatric otorhinolaryngology, AI was described as promising for theoretical learning and standardized clinical scenarios [55]. Beyond knowledge acquisition, emerging evidence suggests a potential role for generative AI in supporting scenario-based and reflective learning. Chatbots were used to explore clinical scenarios [22], while LLMs may function as scalable, case-sensitive reflective prompts rather than authoritative guides, encouraging trainees to reconsider operative plans and reflect on case-specific decisions [59]. Finally, emerging modalities extend educational support beyond text-based interaction. Multimodal systems such as Gemini integrated explanatory images and links into responses, providing additional study resources [53]. AI avatar–based educational platforms enabled rapid production of standardized educational content, reduced faculty workload, and facilitated swift revisions when guidelines changed [54]. Taken together, generative AI was positioned as a multifaceted educational adjunct that can broaden access to specialty knowledge, streamline information retrieval, support reasoning and scenario-based learning, provide personalized feedback and remediation, and extend educational delivery through multimodal and scalable formats. Importantly, its educational role is consistently framed as complementary to established learning resources and expert instruction rather than as a substitute for them [49,50,60].

#### Theme 2: Learning Outcomes, Educational Effectiveness, and Pedagogical Value

The most direct evidence regarding learning outcomes comes from interventional studies embedding generative AI into structured, competence-adjusted educational systems. In the Multi-Expert Knowledge-Aggregated Adaptive Assessment Scheme (MEKAS) study, trainees receiving AI-assisted, competence-adjusted instruction demonstrated significantly greater improvements in clinical core medical knowledge in otolaryngology compared with those undergoing traditional education. The undergraduate- and postgraduate-year (UPGY) experimental group showed markedly higher score gains than controls, and similar benefits were observed in ENT residents, with no significant difference in educational effects between learner groups [41]. Beyond formal knowledge testing, an AI avatar–delivered ENT induction program significantly improved junior physicians’ self-reported confidence in areas including endoscopic anatomy recognition and clinical triage, although acceptability varied [54]. Together, these interventions demonstrated objective knowledge gains and improved learner confidence.

Educational effects were also observed in higher-order cognitive processing and clinical planning. In operative-planning scenarios, LLM-generated feedback altered residents’ clinical decision-making and surgical plans [59]. Rather than functioning as a decision substitute, the model served as a reflective aid, prompting learners to compare their plans with AI-generated alternatives [59]. This “reflection loop” suggests a pedagogical role beyond factual knowledge transfer, supporting structured reasoning, self-evaluation, and reflective practice. A second major line of evidence concerns the quality of AI-generated learning materials. LLMs, particularly GPT-4, generated ENT study guides with high accuracy, relevance, and clarity, closely aligned with residents’ curricular needs and judged by experts as legitimate educational resources [51]. ChatGPT was also considered potentially useful for problem-solving, critical thinking, and reading comprehension [43].

Importantly, pedagogical value was observed even where summative examination performance was limited. Although ChatGPT-3.5 would likely not have passed the American Board of Otolaryngology Written Question Exam, it provided well-constructed explanations and achieved performance comparable to the lower percentile of otolaryngology residents, suggesting educational benefit through explanation, clarification, and conceptual reinforcement [37]. Thus, educational value may extend beyond pass-fail performance to explanatory quality. AI-generated responses in theoretical and clinical scenarios could achieve quality scores comparable to human specialists [55], while ChatGPT-4o showed examination performance statistically comparable to that of ENT residents in 1 study [57].

Overall, generative AI showed potential to support knowledge acquisition, learner confidence, reflective reasoning, and high-quality educational content [37,41-43,51,54-57,59]. However, the current evidence remains stronger for short-term performance, confidence, and process-oriented outcomes than for sustained trainee-level educational benefit. Further studies are needed to determine whether these promising effects translate into durable gains in learning, clinical reasoning, and real-world training outcomes [40]. Current findings favor structured, pedagogically guided use over unguided standalone application.

#### Theme 3: Model Performance, Accuracy, and Variability

Overall, the LLMs achieved moderate to high levels of accuracy on otolaryngology-related tasks, although performance varied substantially across datasets and evaluation settings. Reported accuracies ranged from modest levels, such as 53% on otolaryngology in-service practice examination questions, to high rates of comprehensive or correct responses in structured settings [23,26]. ChatGPT achieved passing-level performance across multiple conditions [24,33]; high accuracy and reproducibility were reported for salivary gland diseases [50]; and newer models performed particularly well in standardized knowledge assessments. For example, o3-mini and GPT-4o achieved accuracies of 93.35% and 90.45%, respectively, on otolaryngology United States Medical Licensing Examination–style questions, significantly exceeding the average medical student performance of 55.44% [61]. High answer-explanation concordance and validity were also reported, particularly for foundational anatomy and pathophysiology [24,25,33,60]. Taken together, current LLMs performed reasonably well on selected ENT knowledge tasks, while remaining strongly dependent on task structure and evaluation context.

A consistent observation across studies was the marked variability in performance across subspecialties, question categories, and difficulty levels [21,48]. LLMs performed better on easier items, whereas more nuanced and in-depth topics posed significant challenges [26]. Similarly, diagnostic tasks were characterized by satisfactory but not comprehensive performance, with models often producing correct answers without consistently providing complete or clinically robust reasoning pathways [31]. Models showed strong performance in theoretical knowledge areas such as cerebrospinal fluid otorrhea, hearing pathophysiology, temporal bone anatomy, and the vestibular system [57] but unsatisfactory performance on auditory physiology questions [22]. Multimodal capability was similarly variable: ChatGPT-4 demonstrated moderately acceptable image-recognition performance [43], while newer GPT-4 models showed substantial improvements on both text- and image-based questions [37]. Thus, accuracy should be interpreted in relation to task complexity, content domain, modality, and reasoning demands, rather than as evidence of generalized competence.

Cross-model comparisons demonstrated rapid generational improvement. Newer models, particularly GPT-4, consistently outperformed GPT-3.5 [37,48]. Standard and custom GPT-4 achieved accuracies of 72% and 81.3%, respectively, compared with approximately 51% to 53% for GPT-3.5 on comparable board-style questions [40], while another study reported 20% improvement in accuracy and 10% improvement in consistency with GPT-4 over GPT-3.5 [22]. In specific domains, such as anatomy learning and examination preparation, GPT-4o achieved particularly high accuracy, exceeding that of DeepSeek, Gemini, and Claude models [62]. However, rankings varied across evaluations: Gemini [48,53] and Bing Copilot [55] were top performers in individual benchmarks, Bard outperformed ChatGPT-3.5 overall and in otology [42], whereas ChatGPT-4 outperformed Bard in skull-base surgery [44]. These rankings reinforce that model superiority is dependent on the comparator, model generation, and evaluation task rather than representing a fixed hierarchy.

Model performance was also multidimensional. Gemini performed well in general otolaryngology question answering and conceptual clarification, whereas GPT-4 was effective for image-based questions and concise, high-accuracy explanations [53]. Claude 2 received the highest ratings for medical adequacy and conciseness, while ChatGPT-4 performed best in covering validated answers [28]. In audiology, ChatGPT generated the most complete responses, whereas Bing Chat performed best for concise relevance [22]. Thus, model performance is multidimensional, and model selection may need to consider the intended educational task and desired response characteristics—including accuracy, completeness, relevance, conciseness, and comprehensibility—rather than overall accuracy alone [22,28].

Variability was also evident in the consistency and stability of model outputs. ChatGPT-4 showed greater consistency than ChatGPT-3.5 [22]. ChatGPT and DeepSeek likewise demonstrated substantial response consistency despite minor fluctuations in an audiologist qualification examination [52], while high reproducibility was reported for ChatGPT responses concerning salivary gland diseases [50]. ChatGPT-5 led a guideline-anchored evaluation in accuracy, comprehensiveness, guideline adherence, and temporal stability [58].

Performance was sensitive to linguistic and structural features of questions. GPT-4 models demonstrated significantly better handling of negated questions than nonnegated ones, whereas Claude 3 Haiku performed poorly on negated questions [48]. Comparable concordance and validity across short-answer and other structured formats further indicated a complex interaction between performance and question structure [25].

Comparisons with human learners and clinicians revealed a more nuanced performance gradient. Earlier evaluations found that LLMs remained inferior to human expertise [21,31], although some were competitive with junior or mid-level residents [35,38]. Conversely, ChatGPT-4 outperformed residents across training years in 1 study, although the performance gap narrowed with increasing residency experience [45], while ChatGPT-4o outperformed residents on selected theoretical knowledge items [57]. Newer models such as o3-mini and GPT-4o also significantly exceeded average medical student performance on standardized knowledge questions [61]. Relative performance of LLMs versus human learners depends substantially on trainee experience, model generation, and the type of competence being assessed; strong performance on knowledge-based questions should therefore not be interpreted as equivalent to specialist-level clinical expertise.

Taken together, contemporary LLMs showed improving accuracy, validity, consistency, and reproducibility, with newer models increasingly reaching or exceeding human learner performance on selected standardized knowledge tasks. However, performance remains heterogeneous across model generations, platforms, subspecialties, modalities, question structures, and reasoning demands. Notably, an accuracy plateau of approximately 73% to 76% among leading general-purpose models has also been reported in specialized otolaryngology knowledge assessment, suggesting persistent limitations without domain-specific adaptation [56]. Thus, high benchmark performance should be interpreted as task-specific capability rather than evidence of uniform competence across the breadth and complexity of otolaryngology [21,24,26,31,37,48,53,56,57,62].

#### Theme 4: Assessment, Examination Readiness, and Question-Type Sensitivity

Across the included studies, the examination performance of LLMs varied substantially according to model generation, assessment format, and task demands, with evidence ranging from suboptimal accuracy to passing or high performance on selected professional examinations. Accuracy in single-answer multiple-choice questions (MCQs) was often 40% to 60% [27], and ChatGPT answered 51% of otolaryngology board-preparation questions correctly [39]. However, advanced models demonstrated stronger performance: GPT-4 passed selected sleep medicine certification questions [29], ChatGPT-4 achieved 75% accuracy and passed the Taiwan Audiologist Qualification Examination [43], and both ChatGPT and DeepSeek exceeded the 60% passing threshold on a standardized audiologist qualification examination [52]. More recent models showed further gains, with o3-mini maintaining high accuracy across difficulty levels and outperforming students, particularly on more challenging questions [61]. Thus, examination readiness has improved with newer models but remains model dependent and task dependent.

A particularly robust and reproducible pattern across studies is the pronounced sensitivity of LLM performance to question format. Multiple evaluations demonstrated significantly poorer performance on MCQs compared with single-choice or open-ended formats. In 1 study, ChatGPT achieved correct responses in more than 60% of single-choice questions but only approximately one-third of MCQs [21]. Multiple-choice underperformance was also statistically significant across all tested models (*P*<.001), suggesting that this limitation reflects a general structural weakness rather than a model-specific deficiency [48]. However, later studies indicate that multiple-choice performance is not uniformly poor. ChatENT reduced errors on MCQs by 26% relative to ChatGPT and outperformed ChatGPT-4 on both multiple-choice and short-answer board questions [25], while DeepSeek and ChatGPT achieved near-passing or passing performance on complex MCQs and high accuracy on reverse-logic questions [52]. Guideline-constrained multiple-choice tasks also produced tightly clustered performance across newer models, suggesting that structured questions anchored to established clinical pathways may reduce response drift [58]. Thus, question-type sensitivity appears to interact with model architecture and task structure rather than representing a fixed limitation of all LLMs.

Question difficulty represents another major determinant of assessment performance. A domain-specific ENT GPT assistant demonstrated a pronounced decline in accuracy from 86.8% on the easiest questions to 42.9% on the most difficult questions [46], whereas o3-mini maintained high performance across all difficulty levels, including 100% accuracy at the greatest difficulty level in 1 evaluation [61]. These findings suggest that sensitivity to difficulty remains model dependent and may diminish as model capabilities advance. Assessment performance was also strongly influenced by modality. GPT-4V performed better on text-only than image-based board examination questions, although providing both textual and visual information improved accuracy for image-based items [36]. GPT-4 performed poorly on direct image interpretation and frequently appeared to infer answers from contextual information rather than the images themselves [45], while Bard was unable to process more than half of image-based questions because of file-processing errors [42]. Graph-based questions were also below the passing threshold [52]. Although newer models showed improvement [42], text-based performance may therefore overestimate competence when visual interpretation is required.

Beyond format and modality, factual knowledge performance did not necessarily reflect higher-order clinical reasoning. Board-style questions may predominantly assess factual recall [38]. ChatGPT-4o performed strongly on theoretical knowledge questions but less well on clinical decision-making, contextual evaluation, and patient-specific judgment [57]. Similarly, general-purpose LLMs performed better on rule-based practice-management content than on clinical subspecialty questions requiring more nuanced reasoning [56]. Narrative case prompts also remained more demanding than guideline-constrained questions, particularly with respect to completeness, stepwise management, and calibration [58]. Thus, strong knowledge-based examination performance should not be equated with clinical reasoning or judgment.

This distinction also has implications for assessment design. Several studies proposed that future examinations should place greater emphasis on higher-order competencies, including diagnostic reasoning, clinical judgment, and image interpretation, rather than relying predominantly on factual knowledge questions that current LLMs increasingly answer successfully [38,45]. ChatGPT-generated otorhinolaryngology MCQs produced correct and unambiguous answer keys in 70% of cases and showed high topic relevance and focus; however, vignette and distractor quality were weaker [34]. AI-assisted question generation therefore remains promising, but expert review remains necessary before formal assessments.

Overall, newer LLMs increasingly achieved passing or high performance on selected examinations but remained sensitive to question format, difficulty, modality, and cognitive demands [36,38,40,42,45,46,50,52,56-58]. They may support examination preparation and formative assessment, but benchmark performance may overestimate real-world competence, particularly when assessments emphasize constrained knowledge tasks [56]. Their improving performance also strengthens the rationale for assessments that explicitly evaluate higher-order reasoning and clinically meaningful competencies.

#### Theme 5: Reliability, Safety, and Risks

A central concern across the included studies was the reliability of generative AI in medical education and clinical knowledge support. Although some evaluations reported high response quality in terms of clarity, relevance, and usefulness, these apparent strengths were consistently undermined by poor source transparency and unreliable citation practices [49,55]. Chatbots produced incorrectly attributed or spurious citations [49], while other studies reported absent source attribution [22] and unreliable or nonexistent references in open-ended responses [32]. Accordingly, successful educational integration requires accurate answers alongside transparent reasoning, reliable sourcing, alignment with curricular goals, and safeguards against outdated or fabricated guidance [60,61].

A major limitation is hallucination. One study reported hallucinated content in more than 13% of open-ended responses, despite apparently coherent and confident outputs [27]. Models also hallucinated during sequential image-based questions, producing similar but incorrect answers across different questions [49]. Additional studies showed that LLMs may produce human-like responses without the contextual reasoning of experienced clinicians [28], may be vulnerable to incorrect or insufficient input information [43], and may generate biased or overly simplified answers when complex medical problems are reduced to fact-based representations [42]. Such errors may be particularly difficult for inexperienced learners to recognize [42].

Safe educational use requires users to retain sufficient biomedical knowledge and critical appraisal skills to recognize inaccurate or oversimplified outputs. One study specifically warned of intellectual dependency and reduced perceived need to learn complex pathophysiology [40]. This concern is particularly relevant for inexperienced learners, supporting the need for expert supervision to prevent uncritical acceptance of confidently presented inaccuracies [26,42,59]. Beyond educational reliability, errors also posed potential patient safety risks. In 1 example, ChatGPT provided incorrect anatomical information regarding surgical planes for an osteoplastic flap, which could compromise outcomes if applied clinically [33]. Several authors cautioned against the routine use of generative AI for clinical decision-making or for unsupervised educational purposes, particularly in contexts where outputs may influence patient care [23,26]. ChatENT reduced safety-related concerns relative to more general-purpose systems [25], suggesting that knowledge grounding and system design can improve—but not eliminate—safety limitations. Residual errors therefore continue to require educator judgment and oversight when generative AI is incorporated into teaching [52].

Reliability concerns extended to AI-generated educational materials. ChatGPT-generated MCQs were generally grammatically correct but sometimes used medical terminology inaccurately or imprecisely, weakening their reliability for formal assessment [34]. The overall quality of such questions was judged to require thorough expert review and revision before use in examinations [34]. These findings reinforce that linguistic fluency should not be equated with domain accuracy and that AI-generated educational content requires specialist validation before curricular or assessment use. Temporal consistency was another reliability dimension. In a 1-month retest of next-generation LLMs, the leading systems preserved their relative performance ranking and demonstrated limited output drift [58]. Thus, reliability should be conceptualized not only in terms of whether an answer is correct but also whether model behavior remains stable, reproducible, and appropriately aligned with established guidance over time.

Generative AI should not be relied on as a stand-alone authority, especially in high-stakes contexts. Multiple studies explicitly advised against overreliance on AI chatbots for final interpretations, assessments, or clinical judgments, emphasizing that such systems should function only as adjuncts to traditional educational resources and expert supervision [23,49]. Even in controlled educational settings, some studies reported that although peer review of AI-generated scripts can mitigate accuracy risks, the lack of interactivity and emotional or pedagogical nuance still limits educational effectiveness and learner acceptance [54]. This adjunctive model is reinforced by newer studies recommending controlled educational use rather than autonomous clinical decision-making [56,57]. In operative-planning education, a supervised “dual-plan” approach—in which residents first generate an independent plan and subsequently compare it with an LLM-generated alternative—was proposed as a way to preserve reflective and metacognitive benefits while reducing the risk of adopting incorrect suggestions uncritically [59]. These findings favor structured workflows that preserve independent reasoning, human judgment, and expert verification.

Overall, current generative AI systems remain constrained by hallucination, unreliable or absent source attribution, susceptibility to flawed inputs, imprecise terminology, residual error, and limitations in contextual and clinical reasoning. Additional risks include intellectual dependency and the uncritical internalization of confidently presented inaccuracies, particularly among inexperienced learners. Although domain-specific grounding and newer model generations may improve safety and temporal stability, they do not remove the need for expert validation and educator oversight. Accordingly, the available evidence supports the use of generative AI as a supervised and transparently governed educational adjunct rather than as an independent source of medical knowledge or clinical decision-making authority [22,25-28,32,34,38,40,42,43,49,52,55-61].

#### Theme 6: Adoption, Usability, and Implementation Barriers

A recurrent facilitator of adoption was ease of use, which influenced users’ willingness to engage with AI-based educational systems. In the MEKAS intervention, both ENT residents and UPGY trainees reported high perceived ease of use scores, supporting usability as an important determinant of acceptance [41]. Similarly, ENTAgents avoided the need for model fine-tuning by using external knowledge bases and self-reflective prompting, thereby lowering technical barriers to deployment [47]. High reproducibility across otolaryngology topics may further support user trust and implementation [23].

Despite these technical advantages, actual adoption among trainees remains limited. Survey data from Italian ENT residents showed modest ChatGPT use, with only a small proportion of trainees having incorporated it into their academic or scientific activities, despite broad awareness of its potential functions [30]. Implementation may also be constrained by unequal institutional access to advanced models and infrastructure, potentially widening disparities in training opportunities [59]. Current use was concentrated on research-related and low-risk academic support tasks rather than core studying. Trainees reported higher perceived usefulness for activities such as citation searching, content searching, and language revision, whereas studying and examination preparation received lower ratings [30]. Generative AI therefore appears to function more as a productivity and academic support tool than as a central learning instrument, possibly reflecting uncertainty about its pedagogical reliability. Larger multicenter studies incorporating diverse assessment formats and updated AI models have been called for to establish safe and effective strategies for integrating generative AI into medical education [57].

Overall, usability, low technical barriers, and reproducibility support implementation feasibility but do not ensure adoption. Wider pedagogical integration requires equitable access, stronger evidence of educational effectiveness across diverse settings, and clearly defined strategies for safe implementation [23,30,41,47,57,59].

#### Theme 7: Advanced AI Architectures and Future Educational Design

A prominent direction in the literature was the shift from general-purpose chatbots toward knowledge-grounded and explainable educational systems. The MEKAS platform exemplifies this approach through a knowledge-based AI design that explicitly prioritizes both accuracy and explainability, supporting learning of clinical core medical knowledge in otolaryngology [41]. This reflects a broader shift toward adapting LLMs to domain-specific knowledge and reasoning requirements rather than relying solely on model scale.

Another trend was the integration of retrieval-augmented generation (RAG) and agent-based frameworks. ENTAgents used agents and RAG to supplement LLMs with up-to-date, specific information from curated knowledge bases and external sources, improving accuracy and completeness [47]. The system also incorporated self-correction and reflective reasoning to refine responses and improve factual grounding [47]. Domain-specific customization also improved specialty performance. ChatENT outperformed ChatGPT-4 on both multiple-choice and short-answer board questions [25], while custom GPT-4 models achieved higher accuracy than standard versions [40]. The ENT GPT assistant demonstrated that customization to specialty-specific language, terminology, and clinical nuances improved contextual relevance and accuracy [46]. Together, these findings favor combining knowledge grounding with specialty customization and expert validation rather than relying on general-purpose models alone [25,28,40,42,46]. In multimodal education, improved medical-image performance may require specialized prompting, advanced image-recognition technologies, and training specifically focused on medical images [36]. Domain refinement, real-time learning mechanisms, and improved interpretability have also been proposed [38].

Generative AI may also support pedagogical designs involving automated generation of case scenarios, differential diagnosis exercises, interactive learning modules, standardized patient simulations, and assessment questions and rubrics, all of which can support scalable, consistent, and resource-efficient instructional design across teaching sessions [49]. Customizable synthetic cases may further expand these applications [29], while virtual-patient scenarios may allow residents and students to practice diagnostic and therapeutic skills in a safe simulation environment [35]. AI has also been proposed for integration into examination simulations that test basic knowledge recall and allow residents to assess gaps in foundational knowledge [45]. In this context, AI is framed not merely as a content generator but as an infrastructure for supporting structured learning workflows, including review, concept reinforcement, board-style question practice, and rapid knowledge recall [49]. Its ability to interact with learners, provide explanations, and respond to user customization also creates opportunities for more interactive and adaptive educational experiences [40].

These systems may further promote active learning and higher-order cognitive skills. AI-generated assessment questions can be used to encourage active engagement rather than passive text review, a strategy that aligns with established principles of memory retention and learning science [49]. At the same time, instructors are encouraged to guide learners to critically appraise AI-generated responses and identify reasoning errors, supporting analytical reasoning, reflective judgment, and diagnostic rigor [49]. In operative-planning tasks, residents were reported to engage with LLM outputs reflectively rather than passively, particularly when AI-generated recommendations differed from their own plans [59]. Future frameworks may therefore promote metacognition by requiring learners to formulate, compare, critique, and revise their reasoning rather than passively accept AI-generated answers [59], while supervised guideline-linked systems may support case discussion and formative assessment [58]. At the architectural level, the literature further suggests that future progress may require explicit support for medical reasoning rather than continued increases in general model scale alone. A reported performance plateau among general-purpose LLMs suggests that adaptation to medical reasoning patterns may represent a more important next step [56]. Hybrid systems combining LLMs with structured knowledge bases or clinical decision trees may provide explicit reasoning frameworks for complex cases [56].

Overall, the evidence suggests a transition from general-purpose conversational AI toward hybrid, domain-adapted, and pedagogically governed educational systems integrating knowledge grounding, retrieval augmentation, specialty customization, structured reasoning, simulation, adaptive interaction, and reflective learning [25,28,29,35,36,38,40-42,45,46,56,58,59]. These approaches position generative AI less as an autonomous answer generator and more as a component of explainable, adaptive, and educationally aligned personalized learning platforms.

## Discussion

### Summary of Evidence

This scoping review suggests that generative AI is increasingly being explored as an educational support tool in otolaryngology. However, the current evidence remains early, heterogeneous, and largely dominated by benchmarking studies rather than direct evaluations of educational effectiveness. Across the included studies, generative AI systems—particularly LLMs—were repeatedly shown to support knowledge retrieval, content synthesis, revision, assessment generation, and scenario-based learning, consistent with broader observations in medical education and clinical informatics [2]. This trajectory mirrors wider developments in digital medicine, where AI is increasingly positioned not merely as an automation tool but as a cognitive and organizational scaffold supporting learning, decision-making, and personalized knowledge pathways [4]. In ENT education specifically, the breadth of subspecialization, anatomical complexity, and heterogeneity of clinical scenarios make the promise of scalable, adaptive, and just-in-time educational support particularly compelling. Importantly, the reviewed literature suggests that personalization extends beyond content adaptation to include immediate feedback, identification of individual knowledge gaps, targeted remediation, self-assessment, and alternative learning pathways, thereby creating opportunities for more adaptive and learner-responsive educational environments, as demonstrated in competence-adjusted educational systems such as MEKAS [41]. Beyond professional education, generative AI is also being explored for patient-facing support in otolaryngology. Studies in postoperative cochlear implant care and tinnitus have reported favorable expert evaluations of AI-generated information, suggesting a potential adjunctive role in supervised patient education [63,64].

Contemporary generative models demonstrated moderate to high performance on structured knowledge tasks, especially in the domains of examination-style questions, anatomy, and guideline-based content. GPT-4 and GPT-4o can reach or exceed passing thresholds and, in some contexts, even outperform junior trainees [45,48]. Similar performance gains have been observed in anatomy education [3]. More recent findings within the reviewed literature further indicate that this human-AI performance relationship is increasingly dependent on the model generation, learner experience, and type of competence being assessed: newer systems may equal or exceed medical students or residents on selected standardized knowledge tasks while continuing to perform less reliably on more complex clinical reasoning tasks [45,57]. Performance therefore remains multidimensional and task dependent rather than reflecting generalized competence [65].

Crucially, this performance heterogeneity is not merely a technical issue but a pedagogical and epistemic one. Performance generally becomes less reliable as tasks require greater complexity, multimodal interpretation, contextual integration, or clinical reasoning, although question format effects are increasingly model dependent rather than uniformly disadvantageous across all contemporary LLMs [26,53]. Thus, strong knowledge-task performance should not be interpreted as a surrogate for expert clinical reasoning, consistent with broader concerns that linguistic fluency may conceal reasoning gaps [8]. This distinction has important implications for assessment design. As LLMs become increasingly capable of answering conventional factual and guideline-based examination questions, traditional knowledge-focused assessments may become less informative measures of uniquely human clinical competence. The reviewed evidence instead supports greater emphasis on diagnostic reasoning, contextual judgment, patient-specific decision-making, image and graph interpretation, and other higher-order competencies [45,57]. Generative AI may therefore influence not only examination preparation but also what medical assessments are designed to measure.

Safety, trustworthiness, and epistemic governance therefore emerge as central concerns. Multiple patient- and clinician-facing studies demonstrate that LLMs continue to hallucinate, misattribute citations, and produce confidently stated but incorrect content [66,67]. An additional risk is intellectual dependency, whereby reliance on AI-generated explanations may reduce the perceived need to master biomedical knowledge, although background knowledge and critical thinking remain necessary to detect erroneous outputs [40]. This problem may be particularly relevant for novice learners, who may simultaneously derive substantial benefit from explanatory support while being less able to detect confidently presented inaccuracies [26,40]. These concerns support assistive, auditable use within frameworks of human oversight [10].

Importantly, the reviewed literature suggests that oversight should function as an active pedagogical strategy rather than merely a final accuracy check. In operative-planning education, residents benefited from comparing their own independently generated plans with LLM-generated alternatives, creating a reflective “dual-plan” process in which disagreement stimulated reconsideration of assumptions and clinical decisions [59]. Structured human-AI interaction may therefore preserve metacognitive benefits while reducing uncritical acceptance of erroneous recommendations [59]. Adoption patterns remain cautious and uneven, with higher acceptance for research assistance and content drafting than for core learning or assessment [1]. Even when AI-based instructional systems demonstrate learning gains [41,68], scalability, generalizability, sustainability, and long-term curricular integration remain largely unresolved. Perceived reliability, demonstrated educational value, institutional guidance, and clearly defined roles within curricula are likely to be equally important [1,41].

Equity is another concern because unequal access to advanced models, infrastructure, and specialist resources may widen disparities in training opportunities [59]. Scalability should therefore not be assumed to imply equitable scalability. An important emerging direction is the shift from stand-alone chatbots toward domain-specific, retrieval-augmented, and knowledge-grounded architectures. Systems such as ChatENT demonstrate that hallucination, inconsistency, and domain drift may be partially mitigated through curated knowledge bases, structured retrieval, and constrained generation [69]. This design philosophy aligns closely with emerging recommendations in trustworthy AI and clinical-grade system development [5]. Specialty-specific customization, agent-based orchestration, self-reflective verification, and structured knowledge representations can further improve contextual relevance and specialty performance, supporting a shift from generic conversational models toward systems explicitly adapted to otolaryngology knowledge and educational objectives [5,25]. This direction may become particularly important as general-purpose LLM performance appears to plateau on specialist knowledge tasks [56]. Hybrid architectures incorporating structured knowledge and reasoning frameworks may therefore represent an important next step, while persistent image-interpretation limitations indicate the need for modality-specific optimization [36,45].

From an educational theory perspective, the greatest long-term impact of generative AI may lie not in passive content delivery but in enabling active learning, formative assessment, adaptive feedback, and reflective practice. AI-generated cases, questions, and study guides already show high perceived clarity and relevance [70], and AI avatars demonstrate the feasibility of scalable, standardized induction training [54]. These applications align closely with modern competency-based education models, in which iterative feedback loops, deliberate practice, and self-regulated learning are more central than static content consumption. The reviewed findings extend this concept by suggesting that the key educational unit may be the human-AI interaction rather than the AI-generated answer itself. When learners are required to formulate, compare, critique, and revise their own reasoning in response to model output, AI may support metacognition and reflective learning rather than merely accelerate information retrieval [59]. This approach is particularly relevant to personalized learning platforms because adaptation can be driven not only by what information learners request but also by their knowledge gaps, errors, reasoning patterns, and responses to feedback [41,59].

At the same time, the literature consistently emphasizes that generative AI cannot be treated as an autonomous epistemic authority. Studies evaluating citation quality, clinical reasoning, and evidence alignment show that expert validation remains indispensable [71,72]. This reinforces a core principle emerging across medical AI: the objective is not simply to automate existing educational activities or replace human educators but to design forms of human-AI collaboration that extend educational reach and responsiveness while preserving independent reasoning, professional judgment, and accountability [5,10,59].

Taken together, the available evidence suggests that generative AI in ENT education remains at an early stage of development. Most studies have focused on technical performance and benchmarking, whereas evidence on educational effectiveness, longitudinal learning outcomes, and real-world curricular implementation remains limited. This scoping review extends the existing literature by moving beyond reviews centered primarily on AI applications or model performance to provide an education-focused synthesis linking generative AI capabilities, learning outcomes, assessment, safety, implementation, and emerging architectures to the design of personalized learning in otolaryngology. Its principal contribution is a conceptual shift from evaluating standalone chatbots toward developing supervised, pedagogically governed, knowledge-grounded, and adaptive learning systems that preserve learner reasoning and expert oversight. This framework has practical implications for educators, curriculum designers, and developers: generative AI can be incorporated into formative assessment, individualized feedback, targeted remediation, and other personalized learning activities, while autonomous or high-stakes use should remain constrained by expert verification and appropriate governance. Accordingly, future research should move beyond benchmark-driven demonstrations toward longitudinal educational outcomes, curriculum-level integration, higher-order reasoning, equitable implementation, and governance addressing safety, transparency, accountability, and human-AI responsibility. This trajectory mirrors the broader evolution of AI in clinical medicine, where early enthusiasm is increasingly balanced by demands for robustness, transparency, and real-world impact [73].

### Limitations

The evidence base has several limitations. First, studies were heterogeneous and predominantly focused on benchmarking and short-term outcomes, with limited longitudinal evidence regarding knowledge retention, transfer to clinical reasoning, or impact on real-world clinical performance. The current literature also provides limited evidence regarding whether repeated use of generative AI influences learners’ independent reasoning, metacognitive skills, or longer-term reliance on external cognitive support. Second, many evaluations rely on artificial test environments or curated question banks, which may not fully capture the cognitive and contextual complexity of authentic clinical learning. This limitation is particularly relevant because the reviewed evidence indicates substantially stronger model performance on constrained, theoretical, or guideline-based tasks than on clinical reasoning, contextual decision-making, and multimodal interpretation. Benchmark accuracy may therefore overestimate educational or clinical competence in real-world settings. Third, the rapid evolution of LLMs means that findings can become partially obsolete within months, limiting the temporal generalizability of performance comparisons between specific model versions. Moreover, differences in access to newer models and supporting infrastructure may limit the generalizability of implementation studies across institutions and may introduce inequities that have received comparatively little empirical investigation.

This review is also constrained by the quality and scope of the available literature. The synthesis is necessarily descriptive and conceptual rather than quantitative, and no formal critical appraisal or effectiveness meta-analysis was possible, given the heterogeneity of study designs. Finally, while we attempted to contextualize ENT-specific findings within the broader AI-in-medical-education discourse, this review did not aim to exhaustively cover the rapidly expanding interdisciplinary literature in this area.

### Conclusions

This scoping review demonstrates that generative AI is evolving from a general-purpose information and content-generation tool toward a broader educational infrastructure capable of supporting knowledge retrieval, formative assessment, immediate feedback, targeted remediation, scenario-based learning, and reflective practice in otolaryngology. Newer models can achieve strong or even human-comparable performance on selected standardized knowledge tasks; however, this capability remains highly dependent on task structure and should not be equated with proficiency in clinical reasoning, contextual judgment, or multimodal interpretation. Persistent risks—including hallucination, unreliable sourcing, confidently incorrect outputs, and learner overreliance—mean that generative AI should remain a supervised educational adjunct rather than an autonomous source of medical knowledge or clinical decision-making authority.

The findings further suggest that the future of AI-enabled personalized learning is unlikely to depend on model scale or answer accuracy alone. More promising approaches combine domain-specific knowledge grounding, retrieval augmentation, structured reasoning support, and pedagogically designed human-AI interaction in which learners remain active participants in generating, comparing, and revising their own reasoning. In this framework, personalization involves not only adapting content but also responding to individual knowledge gaps, errors, and learning trajectories while preserving independent judgment and metacognition. As AI becomes increasingly proficient at conventional knowledge-based assessment, educational research should also consider whether assessment strategies need to place greater emphasis on higher-order reasoning, clinical judgment, and multimodal competence. Future studies should therefore prioritize longitudinal learning outcomes, real-world curricular integration, equitable implementation, and governance models that address transparency, accountability, safety, and the appropriate division of responsibility between learners, educators, and AI systems.

## Supplementary material

10.2196/91785 Multimedia Appendix 1 Generative AI for personalized learning platforms in otolaryngology–head and neck surgery education: scoping review protocol.

10.2196/91785 Multimedia Appendix 2 Full search strategy.

10.2196/91785 Multimedia Appendix 3 Summary of included studies and identified themes (N=42).

10.2196/91785 Multimedia Appendix 4 Major themes identified, associated subthemes, and representative quotations.

10.2196/91785 Checklist 1 PRISMA-ScR checklist.

## Abbreviations

ENT: ear, nose and throat
LLM: large language model
MCQ: multiple-choice question
MEKAS: Multi-Expert Knowledge-Aggregated Adaptive Assessment Scheme
NCBI: National Center for Biotechnology Information
PRISMA-S: Preferred Reporting Items for Systematic Reviews and Meta-Analyses literature search extension
PRISMA-ScR: Preferred Reporting Items for Systematic Reviews and Meta-Analyses extension for Scoping Reviews
RAG: retrieval-augmented generation
UPGY: undergraduate- and postgraduate-year medical students
