# Supplementary material for: Generative AI for Personalized Learning Platforms in Otolaryngology–Head and Neck Surgery Education: Scoping Review
Source: JMIR Med Educ. 2026 Oct 19;12:e91785. doi: 10.2196/91785 (PMC13679907; doi:10.2196/91785)
Supplement: Supplementary file 1 [file mededu-v12-e91785-s001.docx]

**Generative AI for Personalized Learning Platforms in Otolaryngology-head and neck surgery (ENT) Education: Scoping Review Protocol**

**Background**

Otolaryngology–head and neck surgery (ENT, Ear, Nose and Throat) is a broad and technically demanding specialty requiring trainees to integrate knowledge across anatomy, oncology, airway management, otology, rhinology, pediatrics, and perioperative care. In many training systems, undergraduate and early postgraduate exposure to ENT remains limited, while increasing clinical demands constrain protected teaching time. These challenges have increased the need for scalable, flexible, and learner-centered approaches that support self-directed and just-in-time learning.

Generative artificial intelligence (AI), particularly large language models (LLMs), has emerged as a potentially relevant technology for personalized medical education. Generative AI systems can provide explanations, synthesize information, answer questions, generate educational content, and support interactive clinical scenarios. However, their probabilistic and model-dependent outputs also raise concerns regarding reliability, transparency, learner dependency, and appropriate oversight.

Existing evidence on generative AI in ENT education remains heterogeneous and is distributed across performance evaluations, educational interventions, surveys, and system-development studies. A scoping review is therefore appropriate to systematically map this emerging evidence base, identify methodological and educational gaps, and inform future development of pedagogically grounded, AI-enabled personalized learning systems.

**Objectives**

The guiding research question for this review was: “What is the current state of evidence regarding the use of generative AI for personalized learning in otolaryngology (ENT) education?”. We aim to:

1. Systematically map the literature on generative AI in professional otolaryngology education.

2. Characterize the educational contexts and applications that have been studied.

3. Summarize how generative AI has been evaluated, including its performance characteristics and educational outcomes.

4. Identify reported benefits, limitations, safety concerns, and implementation barriers.

5. Synthesize emerging directions relevant to pedagogically grounded, AI-enabled personalized learning platforms in otolaryngology education.

The review is conceptualized around generative AI–enabled personalized learning as the central concept, professional otolaryngology education as the context, and learners, educators, assessment tasks, and educational systems as the relevant units of inquiry.

**Methods**

The review will be conducted and reported in accordance with the PRISMA-ScR (Preferred Reporting Items for Systematic Reviews and Meta-Analyses extension for Scoping Reviews) guidance. Search reporting will additionally follow PRISMA-S guidance.

**Eligibility Criteria**

Eligible sources will include English-language original empirical studies, preprints, and technical or developmental evaluations with direct relevance to professional otolaryngology education.

Studies will be eligible if they:

1. Directly evaluate or apply generative AI or LLMs in professional ENT education or training; and

2. Report empirical data, performance evaluation, user studies, educational interventions, or developmental evaluation.

Review articles, commentaries, expert perspectives, letters, conference abstracts, purely theoretical or technical papers without a direct educational component, and opinion pieces without empirical or developmental content will be excluded. Studies focused primarily on patient education rather than professional education or training will also be excluded.

Publications from 2017 through 2026 will be considered.

**Information Sources and Search Strategy**

The following seven databases will be searched:

1. PubMed (NCBI)

2, Embase (Elsevier)

3. Web of Science (Clarivate)

4. Scopus (Elsevier)

5. ERIC (Institute of Education Sciences)

6. IEEE Xplore

7. CINAHL (EBSCOhost)

Each database will be searched separately. The search strategy will combine controlled vocabulary and free-text terms related to otolaryngology, head and neck surgery, artificial intelligence, generative AI, large language models, ChatGPT, DeepSeek, education, training, personalized learning, teaching, and assessment.

Searches will be restricted to English-language publications from 2017 through 2026. No published search filters or search strategies adapted from previous reviews will be used.

Backward and forward citation searching of included studies will be performed to identify additional potentially eligible sources. No study registries or additional online or print sources will be purposefully searched, and authors or experts will not be contacted to identify additional studies.

The final database search was conducted on July 6, 2026. Complete database-specific search strategies are provided in Multimedia Appendix 2.

**Selection of Sources of Evidence**

All retrieved records will be imported into EndNote 20 and deduplicated using its automated duplicate-detection function.

Two reviewers will independently screen titles and abstracts and subsequently assess potentially eligible full-text reports against the predefined eligibility criteria. Disagreements will be resolved through discussion and, when necessary, adjudication by a third reviewer.

The study-selection process will be documented using the official PRISMA 2020 flow diagram.

**Data Charting Process**

Data will be charted using a standardized form developed iteratively by the review team. Two reviewers will independently chart data from the included sources. Discrepancies will be resolved through discussion or adjudication by a third reviewer.

**Data Items**

For each included study, the following information will be charted:

1. publication year;

2. country;

3. study type;

4. study purpose;

5. setting and educational context;

6. generative AI system evaluated or used;

7. sample size or evaluation set; and

8. findings relevant to the thematic synthesis.

Study types will be assigned using author-reported design terminology when available. When no explicit design label is provided, classification will be based on the study's primary objective and methodological approach. For summary-level reporting, studies will be mapped to five categories: performance study, educational intervention study, system development study, survey study, and guideline development study. This classification is consistent with the final manuscript.

**Critical Appraisal**

Because the purpose of this scoping review is to map the extent, characteristics, and thematic structure of the available evidence rather than estimate intervention effectiveness, no formal critical appraisal or risk-of-bias assessment will be conducted.

**Synthesis of Results**

The extracted evidence will be synthesized using a three-stage inductive thematic approach. Two reviewers will independently examine and code study findings, consolidate related codes into subthemes, and organize these subthemes into broader thematic domains. A third reviewer will contribute to refinement and adjudication where necessary.

The synthesis will focus on educational applications and outcomes, model performance and assessment, reliability and safety, implementation, and emerging educational system architectures. Because substantial heterogeneity is anticipated in study designs, AI models, evaluation tasks, and outcome measures, no quantitative meta-analysis is planned.

**Anticipated Outcomes**

The review is expected to provide a structured map of the current evidence on generative AI in professional otolaryngology education. It will identify areas in which evidence is concentrated, distinguish technical model performance from demonstrated educational effectiveness, and highlight gaps in educational interventions, longitudinal learning outcomes, higher-order reasoning, curriculum integration, real-world implementation, and governance.

The findings are intended to inform educators, curriculum designers, researchers, and developers regarding the potential and limitations of generative AI and to provide a conceptual foundation for future pedagogically grounded, supervised, and knowledge-grounded personalized learning systems.

**Dissemination and Ethics**

The findings will be disseminated through peer-reviewed publication and academic presentation. Because the review synthesizes publicly available literature and does not involve human participants or identifiable personal data, formal ethical approval is not required.

**Conclusion**

This protocol describes a systematic and transparent approach for mapping evidence on generative AI in professional otolaryngology education. The review is intended to clarify the current evidence base and identify priorities for future research on safe, supervised, and pedagogically grounded AI-enabled personalized learning.
