# Supplementary material for: Generative AI for Personalized Learning Platforms in Otolaryngology–Head and Neck Surgery Education: Scoping Review
Source: JMIR Med Educ. 2026 Oct 19;12:e91785. doi: 10.2196/91785 (PMC13679907; doi:10.2196/91785)
Supplement: Supplementary file 2 [file mededu-v12-e91785-s002.docx]

**Multimedia Appendix 2: Full Search Strategy**

1. PubMed (including MEDLINE)

(("Artificial Intelligence"[Mesh] OR "Natural Language Processing"[Mesh] OR "generative artificial intelligence"[tiab] OR "generative AI"[tiab] OR GenAI[tiab] OR "generative model"[tiab] OR "generative models"[tiab] OR "foundation model"[tiab] OR "foundation models"[tiab] OR "large language model"[tiab] OR "large language models"[tiab] OR LLM[tiab] OR LLMs[tiab] OR "multimodal large language model"[tiab] OR "multimodal large language models"[tiab] OR "vision-language model"[tiab] OR "vision-language models"[tiab] OR "vision language model"[tiab] OR "vision language models"[tiab] OR "conversational artificial intelligence"[tiab] OR "conversational AI"[tiab] OR "AI chatbot"[tiab] OR "AI chatbots"[tiab] OR "generative pre-trained transformer"[tiab] OR "generative pre-trained transformers"[tiab] OR "generative pretrained transformer"[tiab] OR "generative pretrained transformers"[tiab] OR "natural language generation"[tiab] OR "retrieval-augmented generation"[tiab] OR "retrieval augmented generation"[tiab] OR ChatGPT[tiab] OR GPT-3[tiab] OR GPT-3.5[tiab] OR GPT-4[tiab] OR GPT-4o[tiab] OR OpenAI[tiab] OR Gemini[tiab] OR "Google Bard"[tiab] OR Claude[tiab] OR Anthropic[tiab] OR Copilot[tiab] OR LLaMA[tiab] OR "Llama 2"[tiab] OR "Llama 3"[tiab] OR Med-PaLM[tiab] OR "Med-PaLM 2"[tiab] OR DeepSeek[tiab] OR DeepSeek-R1[tiab] OR DeepSeek-V3[tiab] OR Grok[tiab] OR Mistral[tiab]) AND ("Otolaryngology"[Mesh] OR "Otorhinolaryngologic Diseases"[Mesh] OR "Otorhinolaryngologic Surgical Procedures"[Mesh] OR "Head and Neck Neoplasms"[Mesh] OR "Audiology"[Mesh] OR otolaryngolog*[tiab] OR otorhinolaryngolog*[tiab] OR "ear nose and throat"[tiab] OR "ear nose throat"[tiab] OR ENT[tiab] OR "head and neck surgery"[tiab] OR "head and neck surgical"[tiab] OR "head and neck oncology"[tiab] OR "head and neck cancer"[tiab] OR "head and neck cancers"[tiab] OR "head and neck neoplasm"[tiab] OR "head and neck neoplasms"[tiab] OR rhinolog*[tiab] OR sinonasal[tiab] OR laryngolog*[tiab] OR otolog*[tiab] OR neurotolog*[tiab] OR audiolog*[tiab] OR "cochlear implant"[tiab] OR "cochlear implants"[tiab] OR "cochlear implantation"[tiab] OR "facial plastic surgery"[tiab] OR "facial reconstructive surgery"[tiab] OR "pediatric otolaryngology"[tiab] OR "paediatric otolaryngology"[tiab] OR "sleep surgery"[tiab] OR "airway surgery"[tiab] OR "skull base surgery"[tiab] OR "sinus surgery"[tiab] OR "thyroid surgery"[tiab] OR "salivary gland surgery"[tiab] OR "parotid surgery"[tiab]) AND ("Education, Medical"[Mesh] OR "Education, Medical, Undergraduate"[Mesh] OR "Education, Medical, Graduate"[Mesh] OR "Education, Medical, Continuing"[Mesh] OR "Teaching"[Mesh] OR "Learning"[Mesh] OR "Curriculum"[Mesh] OR "Computer-Assisted Instruction"[Mesh] OR "Simulation Training"[Mesh] OR "Educational Measurement"[Mesh] OR "Students, Medical"[Mesh] OR "Internship and Residency"[Mesh] OR "Clinical Competence"[Mesh] OR educat*[tiab] OR teach*[tiab] OR learn*[tiab] OR train*[tiab] OR instruction*[tiab] OR curricul*[tiab] OR student*[tiab] OR learner*[tiab] OR trainee*[tiab] OR resident*[tiab] OR fellow*[tiab] OR educator*[tiab] OR faculty[tiab] OR assessment*[tiab] OR evaluat*[tiab] OR examin*[tiab] OR "board exam"[tiab] OR "board exams"[tiab] OR "board examination"[tiab] OR "board examinations"[tiab] OR "in-training examination"[tiab] OR "in-training examinations"[tiab] OR "question answering"[tiab] OR "multiple-choice question"[tiab] OR "multiple-choice questions"[tiab] OR "multiple choice question"[tiab] OR "multiple choice questions"[tiab] OR MCQ[tiab] OR MCQs[tiab] OR feedback[tiab] OR grading[tiab] OR scoring[tiab] OR competenc*[tiab] OR "clinical reasoning"[tiab] OR "personalized learning"[tiab] OR "personalised learning"[tiab] OR "personalized education"[tiab] OR "personalised education"[tiab] OR "adaptive learning"[tiab] OR "individualized learning"[tiab] OR "individualised learning"[tiab] OR "self-directed learning"[tiab] OR "self-regulated learning"[tiab] OR e-learning[tiab] OR elearning[tiab] OR "electronic learning"[tiab] OR "online learning"[tiab] OR "online education"[tiab] OR "distance learning"[tiab] OR "blended learning"[tiab] OR "digital learning"[tiab] OR "intelligent tutoring system"[tiab] OR "intelligent tutoring systems"[tiab] OR "virtual tutor"[tiab] OR "virtual tutors"[tiab] OR "AI tutor"[tiab] OR "AI tutors"[tiab] OR simulation*[tiab] OR "virtual patient"[tiab] OR "virtual patients"[tiab] OR "case-based learning"[tiab] OR "problem-based learning"[tiab]))

2. Embase via Embase.com

(('artificial intelligence'/exp OR 'natural language processing'/exp OR 'generative artificial intelligence':ti,ab,kw OR 'generative ai':ti,ab,kw OR genai:ti,ab,kw OR 'generative model':ti,ab,kw OR 'generative models':ti,ab,kw OR 'foundation model':ti,ab,kw OR 'foundation models':ti,ab,kw OR 'large language model':ti,ab,kw OR 'large language models':ti,ab,kw OR llm:ti,ab,kw OR llms:ti,ab,kw OR 'multimodal large language model':ti,ab,kw OR 'multimodal large language models':ti,ab,kw OR 'vision language model':ti,ab,kw OR 'vision language models':ti,ab,kw OR 'conversational artificial intelligence':ti,ab,kw OR 'conversational ai':ti,ab,kw OR 'ai chatbot':ti,ab,kw OR 'ai chatbots':ti,ab,kw OR 'generative pre trained transformer':ti,ab,kw OR 'generative pre trained transformers':ti,ab,kw OR 'generative pretrained transformer':ti,ab,kw OR 'generative pretrained transformers':ti,ab,kw OR 'natural language generation':ti,ab,kw OR 'retrieval augmented generation':ti,ab,kw OR chatgpt:ti,ab,kw OR 'gpt 3':ti,ab,kw OR 'gpt 3.5':ti,ab,kw OR 'gpt 4':ti,ab,kw OR gpt4o:ti,ab,kw OR openai:ti,ab,kw OR gemini:ti,ab,kw OR 'google bard':ti,ab,kw OR claude:ti,ab,kw OR anthropic:ti,ab,kw OR copilot:ti,ab,kw OR llama:ti,ab,kw OR 'llama 2':ti,ab,kw OR 'llama 3':ti,ab,kw OR 'med palm':ti,ab,kw OR 'med palm 2':ti,ab,kw OR deepseek:ti,ab,kw OR 'deepseek r1':ti,ab,kw OR 'deepseek v3':ti,ab,kw OR grok:ti,ab,kw OR mistral:ti,ab,kw) AND ('otorhinolaryngology'/exp OR 'otorhinolaryngologic disease'/exp OR 'head and neck surgery'/exp OR 'head and neck tumor'/exp OR 'audiology'/exp OR otolaryngolog*:ti,ab,kw OR otorhinolaryngolog*:ti,ab,kw OR 'ear nose and throat':ti,ab,kw OR 'ear nose throat':ti,ab,kw OR ent:ti,ab,kw OR 'head and neck surgery':ti,ab,kw OR 'head and neck surgical':ti,ab,kw OR 'head and neck oncology':ti,ab,kw OR 'head and neck cancer':ti,ab,kw OR 'head and neck cancers':ti,ab,kw OR 'head and neck neoplasm':ti,ab,kw OR 'head and neck neoplasms':ti,ab,kw OR rhinolog*:ti,ab,kw OR sinonasal:ti,ab,kw OR laryngolog*:ti,ab,kw OR otolog*:ti,ab,kw OR neurotolog*:ti,ab,kw OR audiolog*:ti,ab,kw OR 'cochlear implant':ti,ab,kw OR 'cochlear implants':ti,ab,kw OR 'cochlear implantation':ti,ab,kw OR 'facial plastic surgery':ti,ab,kw OR 'facial reconstructive surgery':ti,ab,kw OR 'pediatric otolaryngology':ti,ab,kw OR 'paediatric otolaryngology':ti,ab,kw OR 'sleep surgery':ti,ab,kw OR 'airway surgery':ti,ab,kw OR 'skull base surgery':ti,ab,kw OR 'sinus surgery':ti,ab,kw OR 'thyroid surgery':ti,ab,kw OR 'salivary gland surgery':ti,ab,kw OR 'parotid surgery':ti,ab,kw) AND ('medical education'/exp OR 'teaching'/exp OR 'learning'/exp OR 'curriculum'/exp OR 'computer assisted instruction'/exp OR 'simulation training'/exp OR 'educational measurement'/exp OR 'medical student'/exp OR 'residency education'/exp OR 'clinical competence'/exp OR educat*:ti,ab,kw OR teach*:ti,ab,kw OR learn*:ti,ab,kw OR train*:ti,ab,kw OR instruction*:ti,ab,kw OR curricul*:ti,ab,kw OR student*:ti,ab,kw OR learner*:ti,ab,kw OR trainee*:ti,ab,kw OR resident*:ti,ab,kw OR fellow*:ti,ab,kw OR educator*:ti,ab,kw OR faculty:ti,ab,kw OR assessment*:ti,ab,kw OR evaluat*:ti,ab,kw OR examin*:ti,ab,kw OR 'board exam':ti,ab,kw OR 'board exams':ti,ab,kw OR 'board examination':ti,ab,kw OR 'board examinations':ti,ab,kw OR 'in training examination':ti,ab,kw OR 'in training examinations':ti,ab,kw OR 'question answering':ti,ab,kw OR 'multiple choice question':ti,ab,kw OR 'multiple choice questions':ti,ab,kw OR mcq:ti,ab,kw OR mcqs:ti,ab,kw OR feedback:ti,ab,kw OR grading:ti,ab,kw OR scoring:ti,ab,kw OR competenc*:ti,ab,kw OR 'clinical reasoning':ti,ab,kw OR 'personalized learning':ti,ab,kw OR 'personalised learning':ti,ab,kw OR 'personalized education':ti,ab,kw OR 'personalised education':ti,ab,kw OR 'adaptive learning':ti,ab,kw OR 'individualized learning':ti,ab,kw OR 'individualised learning':ti,ab,kw OR 'self directed learning':ti,ab,kw OR 'self regulated learning':ti,ab,kw OR 'e learning':ti,ab,kw OR elearning:ti,ab,kw OR 'electronic learning':ti,ab,kw OR 'online learning':ti,ab,kw OR 'online education':ti,ab,kw OR 'distance learning':ti,ab,kw OR 'blended learning':ti,ab,kw OR 'digital learning':ti,ab,kw OR 'intelligent tutoring system':ti,ab,kw OR 'intelligent tutoring systems':ti,ab,kw OR 'virtual tutor':ti,ab,kw OR 'virtual tutors':ti,ab,kw OR 'ai tutor':ti,ab,kw OR 'ai tutors':ti,ab,kw OR simulation*:ti,ab,kw OR 'virtual patient':ti,ab,kw OR 'virtual patients':ti,ab,kw OR 'case based learning':ti,ab,kw OR 'problem based learning':ti,ab,kw))

3. Web of Science

TS=(("generative artificial intelligence" OR "generative AI" OR GenAI OR "generative model*" OR "foundation model*" OR "large language model*" OR LLM OR LLMs OR "multimodal large language model*" OR "vision-language model*" OR "vision language model*" OR "conversational artificial intelligence" OR "conversational AI" OR "AI chatbot*" OR "generative pre-trained transformer*" OR "generative pretrained transformer*" OR "natural language generation" OR "retrieval-augmented generation" OR "retrieval augmented generation" OR ChatGPT OR GPT-3 OR GPT-3.5 OR GPT-4 OR GPT-4o OR OpenAI OR Gemini OR "Google Bard" OR Claude OR Anthropic OR Copilot OR LLaMA OR "Llama 2" OR "Llama 3" OR Med-PaLM OR "Med-PaLM 2" OR DeepSeek OR DeepSeek-R1 OR DeepSeek-V3 OR Grok OR Mistral) AND (otolaryngolog* OR otorhinolaryngolog* OR "ear nose and throat" OR "ear nose throat" OR ENT OR "head and neck surg*" OR "head and neck oncolog*" OR "head and neck cancer*" OR "head and neck neoplasm*" OR rhinolog* OR sinonasal OR laryngolog* OR otolog* OR neurotolog* OR audiolog* OR "cochlear implant*" OR "facial plastic surg*" OR "facial reconstructive surg*" OR "pediatric otolaryngology" OR "paediatric otolaryngology" OR "sleep surg*" OR "airway surg*" OR "skull base surg*" OR "sinus surg*" OR "thyroid surg*" OR "salivary gland surg*" OR "parotid surg*") AND (educat* OR teach* OR learn* OR train* OR instruction* OR curricul* OR student* OR learner* OR trainee* OR resident* OR fellow* OR educator* OR faculty OR assessment* OR evaluat* OR examin* OR "board exam*" OR "in-training examination*" OR "in training examination*" OR "question answering" OR "multiple-choice question*" OR "multiple choice question*" OR MCQ OR MCQs OR feedback OR grading OR scoring OR competenc* OR "clinical reasoning" OR "personalized learning" OR "personalised learning" OR "personalized education" OR "personalised education" OR "adaptive learning" OR "individualized learning" OR "individualised learning" OR "self-directed learning" OR "self-regulated learning" OR e-learning OR elearning OR "electronic learning" OR "online learning" OR "online education" OR "distance learning" OR "blended learning" OR "digital learning" OR "intelligent tutoring system*" OR "virtual tutor*" OR "AI tutor*" OR simulation* OR "virtual patient*" OR "case-based learning" OR "problem-based learning"))

4. Scopus

TITLE-ABS-KEY(("generative artificial intelligence" OR "generative AI" OR GenAI OR "generative model*" OR "foundation model*" OR "large language model*" OR LLM OR LLMs OR "multimodal large language model*" OR "vision-language model*" OR "vision language model*" OR "conversational artificial intelligence" OR "conversational AI" OR "AI chatbot*" OR "generative pre-trained transformer*" OR "generative pretrained transformer*" OR "natural language generation" OR "retrieval-augmented generation" OR "retrieval augmented generation" OR ChatGPT OR GPT-3 OR GPT-3.5 OR GPT-4 OR GPT-4o OR OpenAI OR Gemini OR "Google Bard" OR Claude OR Anthropic OR Copilot OR LLaMA OR "Llama 2" OR "Llama 3" OR Med-PaLM OR "Med-PaLM 2" OR DeepSeek OR DeepSeek-R1 OR DeepSeek-V3 OR Grok OR Mistral) AND (otolaryngolog* OR otorhinolaryngolog* OR "ear nose and throat" OR "ear nose throat" OR ENT OR "head and neck surg*" OR "head and neck oncolog*" OR "head and neck cancer*" OR "head and neck neoplasm*" OR rhinolog* OR sinonasal OR laryngolog* OR otolog* OR neurotolog* OR audiolog* OR "cochlear implant*" OR "facial plastic surg*" OR "facial reconstructive surg*" OR "pediatric otolaryngology" OR "paediatric otolaryngology" OR "sleep surg*" OR "airway surg*" OR "skull base surg*" OR "sinus surg*" OR "thyroid surg*" OR "salivary gland surg*" OR "parotid surg*") AND (educat* OR teach* OR learn* OR train* OR instruction* OR curricul* OR student* OR learner* OR trainee* OR resident* OR fellow* OR educator* OR faculty OR assessment* OR evaluat* OR examin* OR "board exam*" OR "in-training examination*" OR "in training examination*" OR "question answering" OR "multiple-choice question*" OR "multiple choice question*" OR MCQ OR MCQs OR feedback OR grading OR scoring OR competenc* OR "clinical reasoning" OR "personalized learning" OR "personalised learning" OR "personalized education" OR "personalised education" OR "adaptive learning" OR "individualized learning" OR "individualised learning" OR "self-directed learning" OR "self-regulated learning" OR e-learning OR elearning OR "electronic learning" OR "online learning" OR "online education" OR "distance learning" OR "blended learning" OR "digital learning" OR "intelligent tutoring system*" OR "virtual tutor*" OR "AI tutor*" OR simulation* OR "virtual patient*" OR "case-based learning" OR "problem-based learning"))

5. ERIC via EBSCOhost

((TI ("generative artificial intelligence" OR "generative AI" OR GenAI OR "generative model*" OR "foundation model*" OR "large language model*" OR LLM OR LLMs OR "multimodal large language model*" OR "vision-language model*" OR "vision language model*" OR "conversational artificial intelligence" OR "conversational AI" OR "AI chatbot*" OR "generative pre-trained transformer*" OR "generative pretrained transformer*" OR "natural language generation" OR "retrieval-augmented generation" OR "retrieval augmented generation" OR ChatGPT OR GPT-3 OR GPT-3.5 OR GPT-4 OR GPT-4o OR OpenAI OR Gemini OR "Google Bard" OR Claude OR Anthropic OR Copilot OR LLaMA OR "Llama 2" OR "Llama 3" OR Med-PaLM OR DeepSeek OR Grok OR Mistral) OR AB ("generative artificial intelligence" OR "generative AI" OR GenAI OR "generative model*" OR "foundation model*" OR "large language model*" OR LLM OR LLMs OR "multimodal large language model*" OR "vision-language model*" OR "vision language model*" OR "conversational artificial intelligence" OR "conversational AI" OR "AI chatbot*" OR "generative pre-trained transformer*" OR "generative pretrained transformer*" OR "natural language generation" OR "retrieval-augmented generation" OR "retrieval augmented generation" OR ChatGPT OR GPT-3 OR GPT-3.5 OR GPT-4 OR GPT-4o OR OpenAI OR Gemini OR "Google Bard" OR Claude OR Anthropic OR Copilot OR LLaMA OR "Llama 2" OR "Llama 3" OR Med-PaLM OR DeepSeek OR Grok OR Mistral)) AND (TI (otolaryngolog* OR otorhinolaryngolog* OR "ear nose and throat" OR "ear nose throat" OR ENT OR "head and neck surg*" OR "head and neck oncolog*" OR "head and neck cancer*" OR "head and neck neoplasm*" OR rhinolog* OR sinonasal OR laryngolog* OR otolog* OR neurotolog* OR audiolog* OR "cochlear implant*" OR "facial plastic surg*" OR "pediatric otolaryngology" OR "paediatric otolaryngology" OR "sleep surg*" OR "airway surg*" OR "skull base surg*" OR "sinus surg*" OR "thyroid surg*" OR "salivary gland surg*" OR "parotid surg*") OR AB (otolaryngolog* OR otorhinolaryngolog* OR "ear nose and throat" OR "ear nose throat" OR ENT OR "head and neck surg*" OR "head and neck oncolog*" OR "head and neck cancer*" OR "head and neck neoplasm*" OR rhinolog* OR sinonasal OR laryngolog* OR otolog* OR neurotolog* OR audiolog* OR "cochlear implant*" OR "facial plastic surg*" OR "pediatric otolaryngology" OR "paediatric otolaryngology" OR "sleep surg*" OR "airway surg*" OR "skull base surg*" OR "sinus surg*" OR "thyroid surg*" OR "salivary gland surg*" OR "parotid surg*")) AND (DE "Artificial Intelligence" OR DE "Medical Education" OR DE "Teaching Methods" OR DE "Learning" OR DE "Educational Technology" OR DE "Computer Assisted Instruction" OR DE "Medical Students" OR DE "Simulation" OR TI (educat* OR teach* OR learn* OR train* OR instruction* OR curricul* OR student* OR learner* OR trainee* OR resident* OR fellow* OR educator* OR faculty OR assessment* OR evaluat* OR examin* OR "board exam*" OR "in-training examination*" OR "question answering" OR "multiple-choice question*" OR "multiple choice question*" OR MCQ OR MCQs OR feedback OR grading OR scoring OR competenc* OR "clinical reasoning" OR "personalized learning" OR "personalised learning" OR "personalized education" OR "personalised education" OR "adaptive learning" OR "individualized learning" OR "individualised learning" OR "self-directed learning" OR "self-regulated learning" OR e-learning OR elearning OR "electronic learning" OR "online learning" OR "online education" OR "distance learning" OR "blended learning" OR "digital learning" OR "intelligent tutoring system*" OR "virtual tutor*" OR "AI tutor*" OR simulation* OR "virtual patient*" OR "case-based learning" OR "problem-based learning") OR AB (educat* OR teach* OR learn* OR train* OR instruction* OR curricul* OR student* OR learner* OR trainee* OR resident* OR fellow* OR educator* OR faculty OR assessment* OR evaluat* OR examin* OR "board exam*" OR "in-training examination*" OR "question answering" OR "multiple-choice question*" OR "multiple choice question*" OR MCQ OR MCQs OR feedback OR grading OR scoring OR competenc* OR "clinical reasoning" OR "personalized learning" OR "personalised learning" OR "personalized education" OR "personalised education" OR "adaptive learning" OR "individualized learning" OR "individualised learning" OR "self-directed learning" OR "self-regulated learning" OR e-learning OR elearning OR "electronic learning" OR "online learning" OR "online education" OR "distance learning" OR "blended learning" OR "digital learning" OR "intelligent tutoring system*" OR "virtual tutor*" OR "AI tutor*" OR simulation* OR "virtual patient*" OR "case-based learning" OR "problem-based learning")))

6. IEEE Xplore

(("All Metadata":"generative artificial intelligence" OR "All Metadata":"generative AI" OR "All Metadata":"GenAI" OR "All Metadata":"large language model" OR "All Metadata":"large language models" OR "All Metadata":"LLM" OR "All Metadata":"LLMs" OR "All Metadata":"foundation model" OR "All Metadata":"conversational AI" OR "All Metadata":"AI chatbot" OR "All Metadata":"ChatGPT" OR "All Metadata":"GPT-4" OR "All Metadata":"GPT-3" OR "All Metadata":"GPT-4o" OR "All Metadata":"transformer model" OR "All Metadata":"retrieval augmented generation" OR "All Metadata":"RAG" OR "All Metadata":"intelligent tutoring system" OR "All Metadata":"AI tutor" OR "All Metadata":"adaptive learning system") AND ("All Metadata":"otolaryngology" OR "All Metadata":"otorhinolaryngology" OR "All Metadata":"ear nose throat" OR "All Metadata":"ENT" OR "All Metadata":"head and neck surgery" OR "All Metadata":"head and neck" OR "All Metadata":"audiology" OR "All Metadata":"laryngology" OR "All Metadata":"rhinology") AND ("All Metadata":"medical education" OR "All Metadata":"surgical education" OR "All Metadata":"education" OR "All Metadata":"teaching" OR "All Metadata":"learning" OR "All Metadata":"training" OR "All Metadata":"simulation" OR "All Metadata":"assessment" OR "All Metadata":"evaluation" OR "All Metadata":"curriculum" OR "All Metadata":"intelligent tutoring" OR "All Metadata":"personalized learning" OR "All Metadata":"adaptive learning" OR "All Metadata":"online learning"))

7. CINAHL via EBSCOhost

((MH "Artificial Intelligence+" OR MH "Natural Language Processing+" OR MH "Machine Learning+" OR TI ("generative artificial intelligence" OR "generative AI" OR GenAI OR "large language model*" OR LLM OR LLMs OR "foundation model*" OR "conversational AI" OR "AI chatbot*" OR ChatGPT OR "GPT-4" OR "GPT-3" OR GPT-4o OR "generative pre-trained transformer*" OR "generative pretrained transformer*" OR "retrieval augmented generation" OR RAG OR Gemini OR Claude OR Copilot OR LLaMA OR Med-PaLM OR DeepSeek OR Grok OR Mistral) OR AB ("generative artificial intelligence" OR "generative AI" OR GenAI OR "large language model*" OR LLM OR LLMs OR "foundation model*" OR "conversational AI" OR "AI chatbot*" OR ChatGPT OR "GPT-4" OR "GPT-3" OR GPT-4o OR "generative pre-trained transformer*" OR "generative pretrained transformer*" OR "retrieval augmented generation" OR RAG OR Gemini OR Claude OR Copilot OR LLaMA OR Med-PaLM OR DeepSeek OR Grok OR Mistral)) AND (MH "Otolaryngology+" OR MH "Head and Neck Surgery+" OR MH "Audiology+" OR MH "Speech-Language Pathology+" OR TI (otolaryngolog* OR otorhinolaryngolog* OR "ear nose throat" OR ENT OR "head and neck surgery" OR rhinolog* OR laryngolog* OR otolog* OR neurotolog* OR audiolog* OR "cochlear implant*" OR "head and neck cancer*" OR "head and neck oncology") OR AB (otolaryngolog* OR otorhinolaryngolog* OR "ear nose throat" OR ENT OR "head and neck surgery" OR rhinolog* OR laryngolog* OR otolog* OR neurotolog* OR audiolog* OR "cochlear implant*" OR "head and neck cancer*" OR "head and neck oncology")) AND (MH "Education, Medical+" OR MH "Education, Continuing+" OR MH "Education, Professional+" OR MH "Teaching Methods+" OR MH "Learning+" OR MH "Curriculum+" OR MH "Simulation+" OR TI (educat* OR teach* OR learn* OR train* OR instruction* OR curricul* OR student* OR trainee* OR resident* OR fellow* OR educator* OR faculty OR assessment* OR evaluat* OR examin* OR "board exam*" OR "in-training examination*" OR MCQ OR MCQs OR feedback OR grading OR scoring OR competenc* OR "clinical reasoning" OR "medical education" OR "surgical education" OR "personalized learning" OR "adaptive learning" OR "self-directed learning" OR e-learning OR elearning OR "online learning" OR "blended learning" OR simulation* OR "intelligent tutoring system*" OR "virtual patient*") OR AB (educat* OR teach* OR learn* OR train* OR instruction* OR curricul* OR student* OR trainee* OR resident* OR fellow* OR educator* OR faculty OR assessment* OR evaluat* OR examin* OR MCQ OR MCQs OR feedback OR grading OR scoring OR competenc* OR "clinical reasoning" OR "medical education" OR "surgical education" OR "personalized learning" OR "adaptive learning" OR "self-directed learning" OR e-learning OR elearning OR "online learning" OR "blended learning" OR simulation* OR "intelligent tutoring system*" OR "virtual patient*")))
