# Supplementary material for: Generative AI for Personalized Learning Platforms in Otolaryngology–Head and Neck Surgery Education: Scoping Review
Source: JMIR Med Educ. 2026 Oct 19;12:e91785. doi: 10.2196/91785 (PMC13679907; doi:10.2196/91785)
Supplement: Supplementary file 3 [file mededu-v12-e91785-s003.docx]

| **Table 1**. Summary of included studies and identified themes (N=42). | | | | | | | |
| --- | --- | --- | --- | --- | --- | --- | --- |
| Study | Country | Study type | Purpose | Setting and context | Generative AI used | Sample Size | Themes |
| Long et al[37], 2023 | Canada | Performance study | Evaluate GPT-4 on OHNSᵃ open-ended board questions using a novel scoring method. | Royal College of Physicians and Surgeons of Canada sample exam. | ChatGPT-4 | 21 open-ended questions with model answers. | MPAVᵇ |
| Hoch et al[20], 2023 | Germany | Performance study | To evaluate ChatGPT accuracy on otolaryngology board certification questions across ENTᶜ subspecialties; To assess performance variability of ChatGPT on practice quizzes for ENT board examination preparation | German ENT board exam question bank | ChatGPT | 2,576 ENT board questions (SCQᵈ + MCQᵉ) | MPAV and AERQSᶠ |
| Revercomb et al[38], 2023 | United States | Performance study | Assess ChatGPT on otolaryngology board-practice questions. | BoardVitals otolaryngology question bank. | ChatGPT | 150 MCQs: 5 per subtopic and difficulty level. | AERQS |
| Zalzal et al[21], 2023 | United States | Survey study | To quantify concordance between ChatGPT and board-certified otolaryngologists on high-level board-style questions | Board-level otolaryngology education | ChatGPT | 60 board-level questions (open-ended + SCQ) | ESLEᵍ, AERQS and RSRʰ |
| Mahajan et al[22], 2023 | United States | Performance study | To evaluate the accuracy and reliability of ChatGPT on ENT residency in-service examination questions | ENT residency in-service exam questions | ChatGPT | 1,088 ENT in-service exam questions | MPAV and RSR |
| Kuşcu et al[23], 2023 | Türkiye | Performance study | To examine the accuracy and reproducibility of ChatGPT responses to HNCⁱ–related questions | HNC information queries | ChatGPT Plus (based on GPT-4, March 13, 2023 version) | 154 HNC questions | ESLE，MPAV，RSR and AUIBʲ |
| Cheong et al[39], 2023 | United Kingdom; Singapore | Performance study | Compare GPT-3.5, GPT-4, and Bard on sleep-medicine board questions. | American Sleep Medicine Certification Board–level questions. | GPT-3.5, GPT-4 and Google Bard | 301 text-based MCQs across 10 categories. | ESLE, AERQS and AAAFEDᵏ |
| Long et al[24], 2024 | Canada (with United States) | Performance study | To assess ChatGPT performance on OHNS certification exams and propose the CVSCˡ evaluation framework | OHNS board short-answer questions | ChatGPT | 21 OHNS board questions (3 trials each) | MPAV and RSR |
| Dallari et al[25], 2024 | Italy | Performance study | To compare ChatGPT responses to ENT clinical cases with specialists for correctness, consistency, and temporal stability | ENT clinical decision-making scenarios | ChatGPT | 20 ENT clinical cases; 3 specialists | ESLE and MPAV |
| Noda et al[25], 2024 | Japan | Performance study | Evaluate GPT-4V on text- and image-based otolaryngology board questions. | 2023 Japanese Otolaryngology Board Certification Examination. | GPT-4V | 100 questions: 54 text-only and 46 image-based. | AERQS, RSR and AAAFED |
| Wang et al[40], 2024 | China (Hong Kong); Taiwan examination context | Performance study | Evaluate ChatGPT-4 on the Taiwan audiologist examination and its educational potential. | 2023 Taiwan Audiologist Qualification Examination. | ChatGPT | 299 MCQs across 6 subjects. | ESLE, LOEEPVᵐ, MPAV, AERQS and RSR |
| Jedrzejczak et al[41], 2024 | Poland | Performance study | Compare ChatGPT, Bing, and Bard on audiological questions. | Defined audiology question set. | ChatGPT; Bing Chat; Google Bard | 10 questions: 3 basic, 3 intermediate, 4 specialized. | ESLE, MPAV and RSR |
| Kochanek et al[42], 2024 | Poland | Performance study | Assess the accuracy and repeatability of ChatGPT-3.5 and GPT-4. | One-year course on objective hearing tests. | ChatGPT | 30 single-answer MCQs. | ESLE, MPAV and RSR |
| Buhr et al[43], 2024 | Germany; United Kingdom | Performance study | Benchmark Bard, Claude 2, and ChatGPT-4 against ORLⁿ specialists. | ORL literature and German state-examination cases. | ChatGPT; Bard; Claude | 41 case-based questions; 8 ORL consultants. | MPAV, RSR and AAAFED |
| Dallari et al[26], 2024 | Italy | Survey study | To assess familiarity, adoption, and perceived usefulness of ChatGPT among Italian ENT residents | Italian ENT residency training survey | ChatGPT | 181 ENT residents (survey respondents) | AUIB |
| Long et al[44], 2024 | Canada; United States | System development study | Develop a cost-efficient specialty LLMᵒ (ChatENT). | Canadian Royal College sample questions and BoardVitals MCQs. | ChatENT; ChatGPT | 23 sample questions; 160 MCQs across 8 sections. | ESLE, MPAV, AERQS, RSR and AAAFED |
| Patel et al[27], 2024 | United States | Performance study | To compare ChatGPT-3.5 and GPT-4 performance on rhinology standardized board examination questions | Rhinology board examination questions | ChatGPT-3.5 and GPT-4 | 127 rhinology board questions (text + image) | LOEEPV and MPAV |
| Terwilliger et al[45], 2024 | United States | Performance study | Compare ChatGPT, GPT-4, and Bard on otolaryngology board questions. | BoardVitals otolaryngology question bank. | ChatGPT; Google Bard | 1,077 text and 60 image-based questions. | LOEEPV, MPAV, AERQS, RSR and AAAFED |
| Su et al[28], 2024 | Taiwan, China | Educational intervention study | To develop and validate a knowledge-based adaptive AI system (MEKASᵖ) for CCMK-OTO^q^ learning | Tertiary-hospital ENT clinical training | Knowledge-based AI (expert-driven AI); ChatGPT used only for grammar correction, not for system function | 54 trainees (UGYʳ, PGYˢ, ENT-Rᵗ); EGᵘ vs CGᵛ | LOEEPV，AUIB and AAAFED |
| Revercomb et al[46], 2024 | United States | Performance study | Compare GPT-4 with GPT-3.5 on otolaryngology board questions. | BoardVitals question bank. | ChatGPT | 150 board-style questions. | LOEEPV, MPAV, AERQS, RSR and AAAFED |
| Lotto et al[47], 2024 | Switzerland; Italy | Performance study | Assess ChatGPT’s ability to generate ORL clinical MCQs. | AI-generated clinical-case quiz administered via Google Forms. | ChatGPT | 20 clinical-case MCQs. | AERQS and RSR |
| Patel et al[48], 2024 | United States | Performance study | Compare ChatGPT with otolaryngology trainees on board-style questions. | Otolaryngology board-style examination. | ChatGPT | 30 questions; 31 medical students and 17 residents. | MPAV, AERQS, RSR and AAAFED |
| Mete et al[49], 2024 | Türkiye | Performance study | Compare ChatGPT, Gemini, Bing, and residents on an ORLITEʷ. | Turkish Society of ORL resident-training textbook. | ChatGPT (GPT-4), Gemini, and Bing | 316 questions; 22 residents (7 junior, 10 mid-level, 5 senior). | ESLE, MPAV and AAAFED |
| Abgin et al[50], 2024 | United States | Performance study | Assess ChatGPT and Bard responses to ICAR:SBˣ policy questions. | ICAR:SB policy statements. | ChatGPT; Google Bard | 34 questions; 2 rhinologists and 2 neurosurgeons. | MPAV |
| Chan et al[29], 2025 | Hong Kong, China | System development study | To develop and evaluate ENTAgents integrating RAGʸ and Reflexion for accurate and explainable ENT knowledge support | ENT knowledge and decision-support tasks | ENTAgent (built on the LangGraph platform); Multi-agent LLM system combining RAG, Reflexion, and tool-augmented agents | 1,035 mixed exam questions (19 SAQᶻ, 24 essay questions, 992 MCQ) | ESLE，AUIB and AAAFED |
| Bicknell et al[51], 2025 | United States | System development study | Develop and evaluate a customized otolaryngology LLM (E-GPT-A). | BoardVitals Otolaryngology and OTOQuest. | ENT GPT Assistant; ChatGPT; Claude | 240 clinical-vignette MCQs. | ESLE, AERQS and AAAFED |
| Hoch et al[30], 2025 | Germany | Performance study | To benchmark advanced LLMs on specialized ENT board questions and assess longitudinal performance changes | German ENT board certification platform | GPT-3.5 Turbo; GPT-4; GPT-4 Turbo; GPT-4o; GPT-4o mini; Gemini 1.0 Pro; Gemini 1.5 Flash; Gemini 1.5 Pro; Claude 3 Haiku; Claude 3.5 Sonnet; Claude 3 Opus | 2,576 ENT board questions; 11 LLMs | ESLE，MPAV and AERQS |
| Trecca et al[31], 2025 | Italy (with Belgium and France) | Performance study | To evaluate the quality of LLM-generated medical information in pediatric otorhinolaryngology | Pediatric ORL education scenarios | ChatGPT-3.5; ChatGPT-4.0; ChatGPT-4o; Claude-3; Gemini; Perplexity; Bing Copilot | 54 pediatric ORL items; clinicians vs LLMs | ESLE，LOEEPV，MPAV and RSR |
| Tassoker et al[32], 2025 | Türkiye | Performance study | To compare accuracy, response time, and answer length of LLMs on anatomy standardized examinations | Head and neck anatomy MCQs | ChatGPT-4o, DeepSeek-v3, Gemini 2.0, Claude 3.7 Sonnet | 74 head and neck anatomy MCQs | MPAV |
| Ramchandani et al[33], 2025 | Canada (with United States and Denmark) | Performance study | To compare performance, response characteristics, and multimodal capabilities of LLMs on ENT question banks | OTO Chautauqua ENT question bank | GPT-4; Microsoft Copilot; Google Bard (early version); Gemini Ultra | 350 ENT MCQs (including image-based) | ESLE and MPAV |
| Patel et al[34], 2025 | United States | Performance study | To assess accuracy, relevance, and clarity of AI-generated study guides for otolaryngology residents | AI-generated ENT study guides | ChatGPT-4 | 4 rhinology chapters; 8 expert reviewers | LOEEPV |
| Prasad et al[52], 2025 | United States | Performance study | Compare GPT-4o and o3-Mini with medical students on otolaryngology questions. | AMBOSS Step 2 CK question bank. | OpenAI | 146 otolaryngology-tagged questions. | ESLE, MPAV, AERQS and RSR |
| Küçüktağ et al[53], 2025 | Türkiye | Performance study | Assess ChatGPT accuracy and reliability for salivary-gland questions. | Questions based on textbooks, guidelines, and ENT expertise. | ChatGPT | 61 questions. | ESLE, MPAV and AERQS |
| Siddique et al[35], 2025 | United Kingdom | Educational intervention study | To evaluate effectiveness, acceptability, and perceptions of an AI avatar–delivered ENT induction course | AI avatar ENT induction training | AI-generated video avatars (non-LLM, content scripted by faculty) | 30 junior doctors (pre-/post-course) | ESLE，LOEEPV and RSR |
| Khoury et al[36], 2025 | United States | Guideline development study | To evaluate AI chatbot performance on head and neck pathology MCQs and inform responsible AI use | Head and neck pathology MCQs | GPT-4o, Claude Sonnet 4, Perplexity, DeepSeek, Open Evidence, Eli5a | 20 pathology MCQs; 6 AI chatbots | ESLE，RSR and AAAFED |
| Ajit-Roger et al[54], 2025 | Canada; United Kingdom | Performance study | Evaluate ChatGPT-4 on Canadian OHNS in-training examinations. | 2022–2023 OHNS National In-Training Exams. | ChatGPT | 2022: 105 residents; 2023: 99 residents. | MPAV, AERQS and AAAFED |
| Qi et al[55], 2025 | China; United States | Performance study | Compare GPT-4 Turbo and DeepSeek-R1 on a Chinese audiologist examination. | 2024 Taiwan Audiologist Qualification Examination. | ChatGPT; DeepSeek | 300 MCQs across 6 subject areas. | ESLE, MPAV, AERQS and RSR |
| Motaghi Niko et al[56], 2026 | Iran | Performance study | Evaluate ChatGPT on Iran’s national pre-internship examination. | March 2022 Iranian pre-internship exam. | ChatGPT | 195 MCQs across 23 specialties. | ESLE, MPAV and RSR |
| Cook et al[57], 2026 | United States | Performance study | Compare GPT-4 Turbo with 10 LLMs on otolaryngology knowledge. | OTOQuest questions from 2020–2022. | GPT-4 Turbo; Claude 3 family; Gemini 1.5 family; GPT-4o; GPT-4o mini; Grok-3 | 1,075 questions; 11 models. | ESLE, LOEEPV, MPAV, AERQS, RSR and AAAFED |
| Günsoy et al[58], 2026 | Türkiye | Performance study | Benchmark five LLMs on guideline-based ENT scenarios. | ENT scenarios based on AAO-HNSᵃᵃ, ERSᵃᵇ, and IFOSᵃᶜ guidelines. | ChatGPT; Gemini; Grok; Llama; DeepSeek | 100 scenarios; 900 questions; 9,000 responses. | MPAV, AERQS, RSR and AAAFED |
| Hack et al[59], 2026 | Israel; United States | Educational intervention study | Assess how LLM assistance affects residents’ operative planning. | Simulated surgical-planning cases across otolaryngology subspecialties. | ChatGPT | 13 residents; 21 scenarios; 267 paired observations. | ESLE, LOEEPV, RSR, AUIB and AAAFED |
| Karatas et al[60], 2026 | Türkiye | Performance study | Compare ChatGPT-4o with ENT residents on a theoretical exam. | ORL specialty-training program. | ChatGPT | 100-item exam; 17 residents. | LOEEPV, MPAV, AERQS, RSR and AUIB |
| ^a^OHNS: Otolaryngology–Head and Neck Surgery  ^b^MPAV: Model Performance, Accuracy, and Variability  ^c^ENT: ears, nose, and throat  ^d^SCQ: single-choice questions  ^e^MCQs: multiple-choice questions  ^f^AERQS: Assessment, Examination Readiness, and Question-Type Sensitivity  ^g^ESLE: Educational Support and Learning Enhancement  ^h^RSR: Reliability, Safety, and Risks  ^i^HNC: head and neck cancer  ^j^AUIB: Adoption, Usability, and Implementation Barriers  ^k^AAAFED: Advanced AI Architectures and Future Educational Design  ^l^CVSC: Concordance, Validity, Safety, Competency  ^m^LOEEPV: Learning Outcomes, Educational Effectiveness, and Pedagogical Value  ^n^ORL: Oto-Rhino-Laryngology/otorhinolaryngology  ^o^LLM: Large Language Models  ^p^MEKAS: multi-expert knowledge-aggregated adaptive assessment scheme  ^q^CCMK-OTO: clinical core medical knowledge in otolaryngology  ^r^UGY: undergraduate-year  ^s^PGY: postgraduate-year  ^t^ENT-R: otolaryngology residents  ^u^EG: experimental group  ^v^CG: control group  ^w^ORLITE: otorhinolaryngology in-service examination  ^x^ICAR:SB: The International Consensus Statement on Endoscopic Skull-Base Surgery 2019  ^y^RAG: retrieval augmented generation  ^z^SAQ: short-answer questions  ^aa^AAO-HNS: American Academy of Otolaryngology–Head and Neck Surgery  ^ab^ERS: European Rhinologic Society  ^ac^IFOS: International Federation of Oto-Rhino-Laryngological Societies | | | | | | | |
