# Supplementary material for: Generative AI for Personalized Learning Platforms in Otolaryngology–Head and Neck Surgery Education: Scoping Review
Source: JMIR Med Educ. 2026 Oct 19;12:e91785. doi: 10.2196/91785 (PMC13679907; doi:10.2196/91785)
Supplement: Supplementary file 4 [file mededu-v12-e91785-s004.docx]

| **Table 3.** Major themes identified, associated subthemes, and representative quotations. | |
| --- | --- |
| **Major theme and subtheme** | **Representative quotation** |
| **Theme 1: Educational Support and Learning Enhancement** | |
| Aiding professional discussion | "In our research into the role of the AI model as an accompanying educational tool for attending Otolaryngologists, we can reject our first null hypothesis, as the system can be used to help in aiding discussion within our field. "[21] |
| Complementary role of AI alongside traditional education | "Use AI chatbots as adjunct tools to complement—not replace—traditional instruction, peer discussion, and textbook/reference-based learning. "[36] |
| Deciphers and interprets intricacies | "ChatGPT was nuanced in answering certain questions from our cohort. Specifically, it was able to understand that questions were related to the field of Otolaryngology, catching the subtlety of ossicle versus auricular that is presented in Table 1, question #2. "[21] |
| Efficient knowledge retrieval and revision support for medical education | "First, ENTAgents can be leveraged to provide medical education to students effectively. With the ability to retrieve solid knowledge from guidelines, books or academic papers in the knowledge base in a second, the learning process can be streamlined, and students can absorb concentrated information efficiently. ENTAgents can also be used in revision for medical students, given its ability to respond to various types of questions comprehensively." [29] |
| High perceived educational value | "The authors believe that utilizing ChatGPT can be an incredibly valuable educational tool for medical students." [25] |
| Potential for rapid medical information retrieval | "With additional validation, ChatGPT or similar tools could serve as invaluable resources for rapid medical information retrieval in high-speed clinical settings, thereby enhancing efficiency and aiding clinicians in their complex decision-making processes." [23] |
| Support for pediatric ORL education and standardized training | "The potential for AI in pediatric ORL is significant, especially for supporting clinicians in theoretical learning and standardized clinical scenarios." [31] |
| Targeted feedback and remediation for independent learning | “In medical education, models like GPT-4o could be employed to simulate board-style questions or provide immediate feedback, fostering independent learning and reinforcing knowledge acquisition. Moreover, by analyzing incorrect responses, LLMs could help trainees identify and address specific knowledge gaps, enabling targeted remediation. ” [30] |
| Efficiency and scalability of AI avatar–generated educational content | "Despite these reservations, the practical benefits of AI avatars are clear. They enable rapid, standardised content production, minimise faculty recording demands, and allow swift updates when guidelines change." [35] |
| Enhanced learning through multimodal explanations in Gemini | "Nevertheless, a unique feature of Gemini was its use of explanatory images and links in answer justifications, which could serve as a study resource. For certain questions, this addition can enhance learning for medical students and residents using the LLM." [33] |
| Bridging sleep-medicine expertise gaps | "As AI begins to revolutionize the field of sleep medicine, addressing both demand pressures and the need for specialized knowledge, it also presents a promising solution to bridge the signifcant gap in sleep medicine expertise, particularly in areas where access to specialists is severely limited."[39] |
| Personalized learning plans | "GPT-4 has the potential to augment the medical education experience by creating personalized academic plans tailored to individual learning styles and ability."[39] |
| Support at junior-resident level | "Gemini and Bing show promise as valuable resources for education and the initial stages of clinical support, as their accuracy levels are similar to junior surgeons."[49] |
| Query-tailored learning | "They provide personalized education by offering responses tailored to specific queries, allowing residents and students to explore complex medical scenarios."[49] |
| Early-residency learning support | "However, they can be preferred for their supportive features in the early years of resident training."[49] |
| Support for learning and awareness | "Furthermore, their ability to mimic human-like responses suggests that they are capable of assisting in educational learning and hearing care awareness."[40] |
| Knowledge and scenario-based learning | "When chatbots are considered for educational or scientific uses, we are thinking of it being used as a source of knowledge or as a test of certain scenarios."[41] |
| Broad clinical and educational support | "Potential applications for clinicians include generating reports, creating document templates, aiding clinical decision-making, simplifying complex information for patient communication, and supporting education"[42] |
| Efficient specialty knowledge retrieval | "A significant advantage of ChatENT lies in its ability to streamline the educational process. It can efficiently provide accurate information, eliminating the need to sift through guidelines, textbooks, or academic papers."[44] |
| Reasoning support for audiology learning | "They also provide a complete reasoning process, enabling teachers and students to quickly grasp key concepts and thought processes, demonstrating their potential as supplementary tools for audiology education."[55] |
| Teaching and information-acquisition potential | "This study suggests that GenAI possesses a certain level of professional knowledge in audiology and has the potential to support teaching and information acquisition."[55] |
| Resident and CME learning support | "The potential utility of text‐based language models in the clinic setting may lie in assisting with educational tools, such as training programs or study resources for residents and clinicians pursuing CME, among other clinical applications."[51] |
| Integration with broader learning resources | "It is deemed important to integrate ChatGPT into a broader learning strategy. Information from AI models should be supported by textbooks, lectures, and training with subject experts. This combination provides a better learning experience and alleviates potential credibility and ethical concerns regarding the use of AI models alone for educational purposes"[53] |
| Rapid feedback and personalized remediation | "Their consistent accuracy across item difficulties suggests real potential as adjunctive learning tools that can deliver rapid feedback, illuminate knowledge gaps, and personalize remediation for medical students preparing for high-stakes exams."[52] |
| Immediate rationales and learning pathways | "For example, AI can provide immediate explanations, detailed rationales, and alternative learning pathways for concepts that students typically find challenging. Such integration can enhance personalized education strategies and address individual learner gaps efﬁciently."[52] |
| Immediate self-assessment feedback | "This means that ChatGPT could be used to help students self‐assess their knowledge by answering past exam questions and receiving immediate, interpretable responses."[56] |
| Support for revision and practice | "While it cannot replace human instruction, its ability to provide accurate answers in specific subjects may support revision and practice in medical education settings."[56] |
| Case-sensitive reflective prompting | "This individualized pattern aligns with the idea of LLMs acting as scalable, case-sensitive reflective prompts rather than as authoritative guides."[59] |
| Basic knowledge study companion | "With 73 to 76% accuracy on board-style questions, these models could serve as effective study companions for residents, particularly for basic knowledge reinforcement and practice management concepts."[57] |
| **Theme 2: Learning Outcomes, Educational Effectiveness, and Pedagogical Value** | |
| AI avatar-delivered ENT induction programme | "This study evaluated an AI avatar-delivered ENT induction programme and found significant improvements in junior doctors’ confidence across multiple domains, with the largest gains in recognising endoscopic anatomy and triaging referrals""[35] |
| Alignment of AI-generated materials with trainee educational needs | "These scores suggest that LLMs are capable of producing material that aligns closely with the educational needs of otolaryngology residents without the need for a field expert to generate these materials." [34] |
| Benefit of competence-adjusted personalized learning | "This outcome suggests that MEKAS effectively assist UPGY in quickly mastering the necessary CCMK-OTO knowledge."; "This finding indicates that competence-adjusted personal learning assistance systems can effectively improve education outcomes, especially for those who are unfamiliar with CCMK-OTO." [28] |
| Comparable benefits across AI-assisted training groups | "The experimental results show that while EG-ENT-R's CCMK-OTO improvement (17.1 %) was numerically lower than the EG-UPGY group's (33.1 %), the difference was not statistically significant (z = −1.659, P = 0.097, r = −0.303). This highlights that both groups benefitted similarly from the training despite the varied improvement rates." [28] |
| Comparable performance to human specialists | "Our analysis revealed that AI models can achieve comparable quality scores to human respondents, particularly specialists, in theoretical and clinical scenarios." [31] |
| Educational value despite lower exam performance of ChatGPT 3.5 | "Although ChatGPT 3.5 likely would not have passed the ABOto WQE, it provided insightful and well‐constructed explanations for the correct answers, and it achieved results consistent with the 8th percentile of otolaryngology residents." [27] |
| High accuracy and clarity of AI-generated study guides | "The study's findings demonstrated that AI-generated study guides can meet high standards of accuracy, relevancy, and clarity, which are essential for medical education. "[34] |
| High-quality study guide generation by GPT-4 | "This study provides compelling evidence that large language models like GPT-4 can generate study guides that are accurate, relevant, and clear, making them a valuable tool in medical education." [34] |
| Improved learner confidence with variable acceptance of AI avatars | "...AI avatar tutors can improve self-reported confidence and are broadly acceptable, though not universally preferred. "[35] |
| Superior learning outcomes with AI-assisted education | "In the present study, the EG-UPGY group achieved a significant improvement in CCMK-OTO compared with the CG-UPGY group. The differences between the scores of the CG, which underwent traditional education, and those of the EG-UPGY groups were pronounced." [28] |
| Supplementary higher-order learning | "Furthermore, ChatGPT's role as a supplementary educational tool in areas requiring aptitude, problem-solving, critical thinking, and reading comprehension has been highlighted."[40] |
| Improved stakeholder learning | "By engaging with AI chatbots, students, teachers, patients, and clinical personnel could significantly improve learning outcomes, patient care, and clinical practice efficiency."[40] |
| Cross-disciplinary educational potential | "GPT-4’s ability to achieve consistently high performance across medical disciplines, including in otolaryngology, supports its potential in the medical education field."[45] |
| Need for trainee-outcome evidence | "Future studies should explore benefits conferred to otolaryngology trainees utilizing AI for medical education."[46] |
| LLM feedback reshapes operative planning | "By systematically analyzing paired responses to structured case scenarios, we show that LLM feedback, delivered via ChatGPT, consistently altered clinical decision-making, led to measurable changes in plan structure, and was perceived by residents as contributing to their planning process."[59] |
| Reflective aid for structured planning | "Our findings extend this by demonstrating real-time influence on upstream cognitive processes. In this sense, LLMs function not as decision replacements, but as reflective aids that guide learners toward more structured, complete plans.This convergence suggests that LLM output may serve as a low-friction mechanism for prompting structured reflection, analogous in function (though not in accuracy) to simulation-based feedback."[59] |
| Measurable changes in surgical plans | "Beyond global impact scores, LLM support led to quantifiable shifts in the structure and content of surgical plans. Residents shortened their responses across multiple domains, particularly in key operative steps and safety protocols."[59] |
| AI-triggered reflection loops | "This finding adds to the literature suggesting that AI-mediated instruction exerts its strongest effects when it triggers a reflection loop: learners compare their own decisions to model outputs, evaluate discrepancies, and re-examine underlying assumptions."[59] |
| Educational value across training levels | "Importantly, no significant differences in perceived utility were observed across PGY levels or subspecialties, indicating that the educational value of the tool was broadly distributed."[59] |
| Comparable performance to ENT residents | "Our study did not find a statistically signiffcant difference between the mean examination performance of ChatGPT-4o and that of ENT residents. These results suggest that artiffcial intelligence-based models may serve as potential supplementary tools in medical education"[60] |
| Stable support for standardized learning | "Their consistent performance across question years indicates stability in core knowledge representation that could support standardized educational applications."[57] |
| **Theme 3: Model Performance, Accuracy, and Variability** | |
| Accuracy for otolaryngology practice exam questions | "Our study reported ChatGPT's overall accuracy to be 53% for a cohort of otolaryngology in‐service practice exam questions." [22] |
| Accuracy variability across otolaryngology subspecialties | "In addition, our analysis included an examination of ChatGPT’s performance across diverse otolaryngology subspecialties, revealing marked variations in the rates of correct responses." [20] |
| Answer easy questions with better proficiency | "Our study differed from those studies in that we stratified questions based on difficulty, showing that ChatGPT may be able to answer easy questions with better proficiency, but more in‐depth, nuanced otolaryngology topics are difficult for the chatbot to correctly answer at this time. "[22] |
| Category–specific performance variability | "The variations in performance among the different otolaryngology categories highlight specific challenges and advantages related to the structure and nature of the questions themselves, as well as the inherent capabilities and limitations of the LLMs." [30] |
| Complementary strengths of Gemini and GPT-4 in ENT education | "Findings from this investigation suggest that Gemini has robust overall capabilities to answer otolaryngology questions or clarify study concepts due to its demonstrated accuracy and adaptability. Additionally, GPT‐4 may be useful for image‐based questions and for learners seeking concise explanations." [33] |
| High accuracy across testing conditions | "In our study, ChatGPT performed well and secured passing scores in all 3 tests: the unprompted test, the first attempt with a prompt, and the regenerated answer with a prompt, scoring 69%, 75%, and 72%, respectively." [24] |
| High accuracy of responses | "Our findings indicate that the majority of responses from ChatGPT were accurate, with 86.4% of receiving a 'comprehensive/correct' rating on our grading scale. Importantly, none of the responses were classified as 'completely inaccurate/irrelevant'." [23] |
| Higher accuracy with concise responses in GPT-4 | "In comparison, GPT‐4, despite shorter responses, demonstrated higher overall accuracy, indicating its effectiveness in providing relevant explanations." [33] |
| Highest accuracy achieved by Gemini | "The analysis of total and subgroup accuracy revealed that Gemini outperformed the other LLMs, followed by GPT‐4, Copilot, and then Bard." [33] |
| Inferior performance compared to human expertise | "These findings suggest that while AI has made considerable advancements, it still falls short of matching the capabilities of its human counterparts." [20] |
| Moderate diagnostic accuracy with limited comprehensiveness | "As evident from our results, Chat-GPT has achieved satisfactory but not excellent levels of correctness and consistency. It demonstrates a good ability to make correct diagnoses in some cases but falls short of providing a comprehensive diagnostic pathway in others. "[25] |
| Moderate performance of Gemini models compared to GPT variants | "The top-performing Gemini model, Gemini 1.0 Pro, achieved an accuracy of 49.1%, which is comparable to Claude 3 Opus but still falls short of the performance exhibited by the GPT variants." [30] |
| Strong but suboptimal performance of Claude models | "Claude 3.5 Sonnet, with an accuracy of 51.9%, and Claude 3 Opus, at 49.1%, demonstrate a robust ability to handle specialized tasks. However, they have not yet reached the top-tier performance levels seen in the GPT-4 variants. "[30] |
| Superior accuracy of ChatGPT-4o for anatomy learning and exam preparation | "ChatGPT-4o demonstrated superior accuracy (98.6%) compared to DeepSeek, Gemini, and Claude 3.7 Sonnet (each scoring 89.2%). This result suggests that ChatGPT-4o may be the most reliable LLM for assisting dental students in anatomy-related learning and exam preparation." [32] |
| Superior handling of negated questions by GPT-4 models | "The analysis of LLM performance on negated versus non-negated questions revealed mixed results across the models."; "Notably, the GPT-4 models demonstrated statistically significant improvements on negated questions compared to non-negated ones. "; "However, the poor performance of Claude 3 Haiku on negated questions highlights that not all LLMs have equally benefited from advancements in linguistic processing." [30] |
| Superior performance of Bing Copilot | "Among the AI models, Bing Copilot emerged as the top performer, underscoring the variability in performance across different platforms." [31] |
| Superior performance of ChatGPT4 | "Meanwhile, ChatGPT4 demonstrated the rapid advancement of AI, boasting a much higher correct response rate on text‐based questions (86.0%) compared to its predecessor (45.2%). It also answered 64.7% of image‐based questions correctly, bringing the overall correct answer rate to 80.3%, still slightly above the average resident score of 76.38% on the total question set." [27] |
| Superior performance of newer AI models | "The results of this study reveal a varied performance landscape among the tested LLMs, with newer or more advanced models, such as GPT-4 and its variants, consistently outperforming their predecessors, including GPT-3.5 Turbo. "[30] |
| The considerable Concordance of Answers Generated by ChatGPT | "During the evaluation, it was observed that the answers provided by ChatGPT were generally concordant (58/63, 92%) and directly addressed the question posed. Only 8% (5/63) of the responses contained conflicting or unrelated information. "[24] |
| The considerable Validity of Answers Generated by ChatGPT | "The majority of the answers provided by ChatGPT were found to be valid: 67% (42/63) were identified as valid, 24% (15/63) were identified as mostly valid, and 10% (6/63) were found to be indeterminate, fair, or mostly invalid." [24] |
| Answer–explanation concordance | "ChatGPT demonstrated considerate concordance, or have explanations that affirm the answer or are directly related to the question"[37] |
| Strong foundational validity | "ChatGPT demonstrated a strong understanding of foundational anatomy and the pathophysiology of OHNS disease presentations. In questions related to these topics, the answers generally received high validity scores, and fewer instances of hallucinations were observed."[37] |
| Consistent passing performance | "In our study, ChatGPT performed well and secured passing scores in all three tests: the unprompted test, the first attempt with a prompt, and the regenerated answer with a prompt, scoring 69.1%, 75.0%, and 72.1%, respectively."[37] |
| Near mid-level resident performance | "Mid-level residents showed superior results with an accuracy of 63.45% compared to Gemini (p<0.001), though ChatGPT approached their performance levels (p=0.013). LLMs were found to be competitive only with junior residents."[49] |
| Greater accuracy of Chat-GPT 4 than Bard | "After evaluation by fellowship-trained rhinologists and neurosurgeons, Chat-GPT 4 demonstrated statistically significant superiority in accuracy over Bard."[50] |
| Moderate image-recognition ability | "ChatGPT-4 demonstrated a moderately acceptable level of image recognition performance, successfully identifying over half of the content within the images"[40] |
| Claude leads adequacy and conciseness | "Of all tested LLMs Claude 2 was rated best in the categories of medical adequacy and conciseness and was only slightly surpassed by ChatGPT 4 for coherence."[43] |
| High overall comprehensibility | "The comprehensibility of answers received the highest overall ratings for all LLMs."[43] |
| Model-specific adequacy strengths | "Although Claude 2 received the highest rating for medical adequacy among the LLMs, ChatGPT 4 performed best in covering the validated answers."[43] |
| ChatGPT provides most complete answers | "The ChatGPT responses were rated as the most complete. However, responses from all chatbots were generally rated as not entirely complete. Speciﬁcally, Bing Chat responses received the lowest score in terms of completeness; ...while ChatGPT is better when searching for more comprehensive information."[41] |
| Bing excels in concise relevance | "In terms of relevance, Bing Chat received the highest score, followed closely by ChatGPT; Bing Chat seems best when searching for short answers, ...while ChatGPT is better when searching for more comprehensive information."[41] |
| Poor auditory-physiology performance | "None of the ChatGPT versions we tested provided satisfactory performance regarding questions on auditory physiology."[41] |
| GPT-4 improves accuracy and consistency | "Nonetheless, ChatGPT 4 surpassed ChatGPT 3.5 in both accuracy and consistency over time, demonstrating a 20% improvement in accuracy and a 10% enhancement in consistency."[41] |
| Stable response variability over time | "Despite these differences, the response variability remained consistent across different time intervals-minutes, days, and a week."[41] |
| Comparable short-answer validity | "Upon evaluation with short‐answer questions (as illustrated in the attached sample questions), the performance of the system exhibited comparable concordance and validity."[44] |
| Bard outperforms ChatGPT-3.5 | "In comparing these models in more depth, Bard achieved higher scores compared to ChatGPT, performing better overall (61.7% vs 55.3%, p<0.01) and in the otology section (65.1% vs 52.6%, p<0.01)."[45] |
| GPT-4 exceeds GPT-3.5 accuracy | "Our study found that standard (72.0%) and custom GPT-4(81.3%) demonstrated higher accuracy than GPT-3.5 on the same 150 questions included in our previous study (51.3%) and on similar Otolaryngology board-style questions (53%)"[46] |
| Performance between junior and senior trainees | "Findings reveal ChatGPT’s superiority over beginners but eventual inferiority to seasoned tolaryngology residents on board-style questions targeting otolaryngology knowledge, indicating a progressive convergence in performance."[48] |
| Performance depends on trainee level | "ChatGPT can outperform lower-level medical trainees on Otolaryngology board-style exam but still lacks the ability to outperform higher-level trainees."[48] |
| Outperforms residents on knowledge questions | "Overall, ChatGPT-4 performed significantly better than residents at all training years (Multimedia Appendices 1 and 2), with a decreasing difference with increasing residency training."[54] |
| Similar overall model capability | "These results indicate that the 2 models exhibited substantial response consistency despite minor fluctuations, suggesting highly similar overall capabilities in the audiologist qualification examination."[55] |
| High accuracy and reproducibility | "In this study, the accuracy and reproducibility of ChatGPT’s answers to questions about salivary gland diseases were found to be acceptably high."[53] |
| AI surpasses average student performance | "Overall, o3-Mini demonstrated the highest accuracy across all questions, achieving an average accuracy of 93.35%, closely followed by GPT-4o with an accuracy of 90.45%. Both AI models significantly outperformed the average medical student performance, which was 55.44% (P=0.008 and P=0.012, respectively)."[52] |
| Performance exceeds human baseline | "This result highlights that current-generation GPT models have reached and surpassed human baseline performance on standardized medical knowledge assessments, consistent with recent literature highlighting AI’s rapidly advancing capabilities in medical education."[52] |
| Outstanding otorhinolaryngology performance | "ChatGPT demonstrated its highest expert rating in the field of otorhinolaryngology (mean = 4.92), indicating outstanding performance in this specialty."[56] |
| ChatGPT-5 leads multidimensional performance | "All models performed within a narrow, clinically credible range, with ChatGPT-5 consistently leading in accuracy, comprehensiveness, guideline adherence, and temporal stability."[58] |
| Narrative reasoning increases variability | "Aligns with broader evaluations in clinical AI, showing that modern LLMs are strongest when tasks are well scaffolded and knowledge is directly retrievable, and comparatively more variable when narrative reasoning and completeness are stressed."[58] |
| Strength in theoretical knowledge | "ChatGPT-4o demonstrated higher performance than residents in knowledge-based items, particularly “Cerebrospinal Fluid Otorrhea,” “Hearing Pathophysiology,” “Anatomy of the Temporal Bone,” and “Vestibular System.” These findings indicate that artiffcial intelligence models have a strong capacity to process theoretical knowledge and may outperform humans in selected domains"[60] |
| General-purpose accuracy plateau | "The observed accuracy plateau of approximately 73 to 76% among the top-performing models suggests that current general-purpose LLMs may be limited in specialized medical knowledge without domain-specific training."[57] |
| **Theme 4: Assessment, Examination Readiness, and Question-Type Sensitivity** | |
| Difficulty in accurately answering multiple-choice questions | "Of note, specifically in single-choice questions, ChatGPT was most successful, with over 60% rate of correct answers. In contrast, multiple-choice questions appeared to be a greater hurdle for ChatGPT: only one third of this question type could be answered correctly. "[20] |
| Inferior performance on multiple-choice questions | "Another critical observation from our study is the consistent underperformance of LLMs on multiple-choice questions compared to single-choice questions, a trend that was statistically significant across all models (p < 0.001)." [30] |
| Insufficient reliability for board examination preparation | "Notably, ChatGPT delivered a considerable number of incorrect responses within specific otolaryngology subdomains, rendering it unreliable as the sole resource for residents preparing for otolaryngology board examination." [20] |
| Limited accuracy in single‐answer multiple‐choice scenario | "This differs from our experience into the matter, albeit on a smaller scale, where ChatGPT in our single‐answer multiple‐choice scenario only answered 43.3% of questions correct."; "Nevertheless, both our study and Hoch et al demonstrated ChatGPT accuracy in the range of 43% to 57%..."[21] |
| Potential in answering multiple‐choice questions | "There is significant potential in AI answering multiple‐choice questions, but at this time, it does not fare as successfully in this task as it does for open‐ended questioning." [21] |
| Board-exam preparation support | "Our study assessed ChatGPT's performance on questions designed for Otolaryngology board examination preparation and found it answered 51% of questions correctly...The performance of ChatGPT in this study suggests it can be used as an additional resource for preparing for Otolaryngology board certiffcation,while maintaining caution about the validity of its responses."[38] |
| Superior sleep-exam performance | "GPT-4 has demonstrated signifcantly superior overall performance compared to Google Bard and the ability to pass certain exam categories of self-assessment questions at the level of the American Sleep Medicine Certifcation Board Exam within a 4-month interval from its previous iteration GPT-3.5."[39] |
| Text-over-image accuracy advantage | "The results confirmed that the accuracy was higher for text-only questions than for image-based questions; Our findings showed that the accuracy of GPT-4V for image-based questions was lower than that for text-only questions."[61] |
| Limited image-analysis capability | "This suggests that, although AI excels at analyzing textual information, it still has limitations in analyzing image-based data"[61] |
| Multimodal input improves accuracy | "GPT-4V demonstrated a certain level of accuracy for the 2023 otolaryngology board ertification examination, and text-plus-image input increased the accuracy of image-based questions."[61] |
| Passing audiology-exam performance | "ChatGPT-4 achieved an overall accuracy rate of 75%, meeting the passing criterion necessary for candidates to obtain the basic qualification for practicing as clinical audiologists in Taiwan."[40] |
| Lower MCQ error rate | "Further, when subjected to MCQs, the system yielded satisfactory outcomes, outperforming ChatGPT with a commendable 26% reduction in errors."[44] |
| Superior board-question performance | "It exhibited superior performance to ChatGPT4.0 when tested on both multiple-choice and short-answer questions sourced from board examination materials."[44] |
| GPT-4 leads across difficulty and modality | "Notably, GPT-4 (78.7%) performed better than Bard (61.7%, p<0.001) and ChatGPT-3.5 (55.3%, p<0.001) across all difficulty levels, on all examination sections, and with questions incorporating images."[45] |
| Persistent image-processing limitations | "This is evidenced when comparing Bard and GPT-4, with GPT-4 scoring 56.7% on the set of 60 image questions and Bard 46.4% on the images it could interpret (p=0.368). Bard was unable to process >50% of image-based questions due to file processing errors."[45] |
| Improved performance on selected question types | "Overall, our study demonstrated improved performance by standard and custom GPT-4, with more correct answers on ‘easy,’ longer, and plastic and reconstructive questions."[46] |
| Mostly correct answer keys | "Overall, we report a correct and unambiguous answer key in 70% of the questions and a mean score of 3.8/5 (SD ± 1.83) for the correct answer quality."[47] |
| Strong relevance but weak distractors | "Overall correct answer quality scored 3.75/5 (SD ± 1.81). Focus quality and topic relevance showed an over‐average score of respectively 4.7/5 (SD ± 0.95) and 4.7/5 (SD ± 0.95). Vignette quality and distractor quality performed worse with a score of 3.1 (SD ± 1.24) and 2.6/ 5 (SD ± 1.2). On the quality assessment, clear wording was analyzed, which was mostly the case."[47] |
| Rote knowledge does not equal clinical reasoning | "These questions primarily test rote memorization of medical facts; in contrast, the art of practicing medicine is predicated on the synthesis of complex presentations of disease and multilayered application of knowledge of the healing process."[48] |
| Poor direct image interpretation | "ChatGPT-4 had a poor performance on image-based questions, frequently appearing to make educated guesses based on the available context provided without directly analyzing the images."[54] |
| Image limitations should shape assessment | "Its poor performance on image-based questions indicates an important limitation of this tool. Ensuring that future examinations address skills that AI systems, such as ChatGPT, cannot yet do efficiently is an area that merits attention."[54] |
| Prioritize higher-order assessment | "As such, developing higher-order thinking testing, including image-based analysis, should be prioritized to adequately measure the required cognitive skills to become a proficient surgeon"[54] |
| Question length has little effect | "Our analysis revealed that question length did not have a significant impact on the chatbot’s performance."[54] |
| Weakness in clinical reasoning skills | "Shifting toward testing diagnostic reasoning, clinical judgment and image interpretation, critical skills for a proficient surgeon, for which ChatGPT demonstrated limited performance, should be strongly considered."[54] |
| Need for higher-order competency testing | "As such, developing higher-order thinking testing, including image-based analysis, should be prioritized to adequately measure the required cognitive skills to become a proficient surgeon."[54] |
| Both models exceed passing threshold | "Both GenAI models achieved test scores exceeding the qualification examination’s passing criterion of 60% accuracy, demonstrating overall strong performance."[55] |
| Weak graph-based performance | "Question-type analysis revealed that both models underperformed on graph-based questions, with DeepSeek scoring 4 out of 11 (36% correct) and ChatGPT scoring 2 out of 11 (18% correct), both below the passing threshold."[55] |
| Near-passing complex-question performance | "For complex multiple-choice questions, both approached the passing level, with DeepSeek scoring 11 out of 17 (65% correct) and ChatGPT scoring 9 out of 17 (53% correct)."[55] |
| Strong reverse-logic performance | "In reverse logic questions, both models performed well at comparable levels, with DeepSeek scoring 80 out of 95 (84% correct) and ChatGPT scoring 79 out of 95 (83% correct)."[55] |
| Accuracy declines with question difficulty | "Another critical finding of this study is the impact of question difficulty on model performance and inconsistency of responses, which further highlights areas for future development. The accuracy of E‐GPT‐A declined significantly from 86.8% for tier 1 (easiest) questions to 42.9% for tier 4 (most difﬁcult) questions."[51] |
| Better performance on basic questions | "Consistent with our findings, ChatGPT performs better on basic questions, whereas its success rate decreases for more complex topics such as treatment management."[53] |
| Training and exam-preparation resource | "ChatGPT serves as an additional source of information that otolaryngology professionals can use for their academic training and exam preparation."[53] |
| High accuracy across difficulty levels | "Regarding performance, o3-Mini consistently maintained high performance across all difficulty levels, even achieving 100% accuracy at the most challenging level. GPT-4o, despite its slightly lower overall performance, still showed remarkable accuracy and outperformed students, particularly at intermediate difficulty levels."[52] |
| Guideline constraints reduce response drift | "Guideline-constrained question design appears to reduce open-ended drift and reward recall of the consensus pathway; our tight inter-model clustering on multiple-choice items suggests that modern LLMs have largely internalized ffrst-line ENT algorithms."[58] |
| Narrative prompts test calibration | "Narrative prompts remain a stress test for chain-of-thought fidelity and calibration; small but durable advantages for the top system (ChatGPT-5) arose from fewer omissions and better articulation of stepwise management, an observation consistent with broader assessments that highlight instructionfollowing and calibration as determinants of clinical utility"[58] |
| Weak clinical and contextual reasoning | "On the other hand, ChatGPT-4o showed limited success in items requiring clinical decision-making, such as “Surgical Approach.” These results suggest that clinical insight, contextual evaluation, and patient-speciffc variables remain challenging for artiffcial intelligence models."[60] |
| Theory strength but decision-making limits | "The results of our study demonstrated that artiffcial intelligence-based models are comparable to, or may even outperform, residents in theoretical knowledge-based examinations. However, the model’s success was limited for some questions requiring clinical and contextual decision-making."[60] |
| Knowledge–reasoning performance gap | "ChatGPT-4o demonstrated performance on a theoretical multiple-choice examination comparable to that of otorhinolaryngology residents, particularly on knowledge-based questions, while showing limitations in domains requiring clinical decision-making and contextual reasoning."[60] |
| Rule-based tasks exceed clinical reasoning | "The superior performance in Practice Management (86.7%) compared with clinical subspecialties like Head and Neck Surgery (66.2%) highlights an important distinction: models appear to handle rule-based, administrative content more effectively than complex clinical reasoning."[57] |
| Foundational mastery but reasoning limits | "The significant performance gradient across question difficulty levels demonstrates that these models have effectively mastered foundational otolaryngology knowledge but struggle with the nuanced reasoning required for more challenging clinical scenarios."[57] |
| Benchmarks may overestimate real-world ability | "While suitable for benchmarking, this evaluation format constrains the problem space and likely overestimates performance compared with open-ended or case-based assessments."[57] |
| **Theme 5: Reliability, Safety, and Risks** | |
| Accuracy ensured by peer review but limited interactivity | " In this study, peer review of scripts mitigated accuracy risks, but the lack of interactivity and emotional nuance likely contributed to mixed perceptions." [35] |
| Avoidance of overreliance on AI for high-stakes decisions | "Limit dependency on AI chatbots for final interpretations or high-stakes assessments; they should not be the sole source of study or knowledge acquisition." [36] |
| Caution against routine clinical or educational use | "At present, we caution against the routine use of AI as a medical education tool or as a means to support resident physician decision‐making if we were to extrapolate the data beyond what is found in the scope of this study." [22] |
| Hallucinating or confabulating | "Another observation discovered during this process was the hallucinating, or confabulating, answers by the LLM.""This event occurred in 13.33% (N = 4) of open‐ended questions." [21] |
| Insufficient readiness for educational implementation | "Furthermore, we argue that this technology also has not advanced enough to be a reliable learning tool for early trainees who may not be able to distinguish an incorrect from a correct answer or explanation."; "...the degree of inaccuracy and the subsequent spurious explanations used to support the incorrect answers could be detrimental to learners..." [22] |
| Multimodal accuracy gains with image-related hallucination risk | “Our results suggest that better accuracy is achieved when diverse visual data is combined together. We also noticed that Perplexity (Perplexity AI) and GPT4o (OpenAI) will hallucinate when image-based questions are prompted immediately after each other resulting in a similar response for different questions...” [36] |
| Not advisable to rely solely | "However, we are only in the early stages of the era of AI-provided medical services, and it is currently not advisable to rely solely on the existing version of ChatGPT as the only source of medical information. "[23] |
| Potential patient safety risks due to inaccurate responses | "However, there are situations where ChatGPT’s inaccuracies could potentially compromise patient safety. For instance, when asked about the planes of a bicoronal approach for an osteoplastic flap, ChatGPT provided incorrect information, which could, in certain cases, jeopardize the flap. "[24] |
| Unreliable citation attribution and risk of overreliance | “As indicated by the results, the citations offered by the chatbots often were not appropriately attributed to the correct answer.”； “Collectively, these results suggest that trainees should avoid overreliance on AI chatbots particularly if they are seeking accurate high quality scientific citations.” [36] |
| High response quality but limited source transparency | "Our results evidenced that AI models demonstrated strong performance in clarity, relevance, and usefulness of their responses. However, they consistently underperformed in providing transparent and reliable sources, which is a key limitation for integrating AI into evidence‐based clinical practice." [31] |
| Need for informed AI use | "This highlights the importance of understanding these limitations and appropriately using AI in medical education and clinical diagnoses within the otolaryngology field."[61] |
| Vulnerability to flawed information | "However, in fields such as electrophysiological audiology, which depend more on precise knowledge points, the accuracy of ChatGPT-4 was challenged when confronted with incorrect or insufficient information."[40] |
| Human-like output without reasoning | "This example illustrates LLM’s potential to generate human-like responses but without the ability to ‘think’ like an experienced human counterpart.Possible explanations for the poor performance in the Weber test may originate in a lack of sufficient training data may result in ‘hallucination’. Alternatively, deficits of LLMs in the detection of context may be attributable."[43] |
| Lack of source transparency | "Currently, any application to science or education is ruled out because reference to sources is not provided, although it is true that in normal conversation one does not usually cite sources."[41] |
| Unreliable or fabricated citations | "This is particularly concerning when ChatGPT generates a narrative or answers an open-ended question without providing reliable sources or, in some cases, citing non-existent references"[42] |
| Reduced safety concerns | "There was a marked reduction in safety‐related issues, which are of paramount concern in health care applications"[44] |
| Bias and hallucination risks | "As a study outlet, AI may skew complex medical problems into more abstract and one-dimensional answers based solely on facts. While the explanation of AI may provide some factual insight into the question at hand, it may also provide false assumptions and biased responses based on the availability of data. It is also important to warn against the phenomenon of AI “hallucinations,” where language models can present erroneous information in a sensible or factual manner"[45] |
| AI as a cautious study adjunct | "While the results of this study suggest AI as an emerging study adjunct, they also suggest caution regarding the use of AI (and especially more primitive AI models, like ChatGPT and Bard) as a primary study tool for medical students and residents."[45] |
| Hallucinations threaten learning safety | "These hallucinations can be challenging to detect, especially for non-medical users or inexperienced medical trainees, and can provide inaccurate medical information that could compromise the educational experience and even patient care."[45] |
| Unreliable complex-problem answers | "This study shows that while current AI models demonstrate promise for use as study adjuncts in otolaryngology, they have not yet provided consistent, reliable answers for complex medical problems that may be on medical board exams."[45] |
| Risk of intellectual dependency | "There is the risk of intellectual dependency on AI and a perceived decrease in the need to learn complex pathophysiology. However, the present state of AI requires biomedical knowledge of diseases and critical thinking to appraise newly developed AI systems"[46] |
| Imprecise terminology reduces reliability | "Nevertheless, according to this panel of questions, ChatGPT does not seem to be able to use the exact medical terms correctly. There were expressions used inappropriately or imprecisely, which weakened the reliability of the exam questions."[47] |
| Expert review required for AI-generated MCQs | "ChatGPT 3.5 is able to generate grammatically correct simple ORL multiple choice questions for a medical student level; however, the overall quality of the questions was average, needing thorough review and revision by a medical expert to ensure suitability in future exams."[47] |
| Need for cautious specialty deployment | "Our findings emphasize the need for caution and meticulous assessment when deploying language models in specialized fields like otolaryngology or medicine, where precision is critical, and the stakes are high."[48] |
| Not a trusted information standard | "Considering this, ChatGPT is not yet intelligent enough to become the trusted gold standard to accessing medical information within Otolaryngology."[48] |
| Educator oversight for residual errors | "GenAI responses still contain a certain error rate, and thus, their use in teaching requires careful judgment and guidance from educators."[55] |
| Safeguarded curricular integration | "Nevertheless, successful educational integration must go beyond answer generation to emphasize transparent reasoning, alignment with curricular goals, and safeguards against hallucinations or outdated guidance."[52] |
| Need for domain accuracy and curricular alignment | "This means that although ChatGPT shows promise as a supplementary tool in medical education, further development is necessary to enhance its domain-specific accuracy, reduce confidently incorrect outputs, and ensure alignment with curriculum standards."[56] |
| Temporal stability signals reliability | "Temporal consistency favored models with stronger adherence and stability proffles; our one-month retest preserved rank order and showed the most negligible drift in the leading systems, echoing the literature’s emphasis on reliability—not raw accuracy alone—for real-world deployment"[58] |
| Oversight preserves metacognitive benefits | "Taken together, these findings emphasize that while LLMs can reshape cognitive organization and prompt reflective reasoning, they require oversight to mitigate propagation of erroneous content.A supervised “dualplan” model, in which residents generate an independent plan before comparing it with an LLM-generated alternative, may preserve the metacognitive benefits while preventing uncritical internalization of inaccuracies."[59] |
| Supervised reflective simulation adjunct | "These findings suggest that generative AI may function as a structured reflective adjunct within supervised simulation settings, but it should not be used as an independent instructional authority."[59] |
| Controlled educational use only | "Large language models such as ChatGPT may be considered educational tools when used in a controlled manner, but they should not be used alone as clinical decision-making tools."[60] |
| Adjunctive rather than autonomous use | "Combined with the observed performance plateau, these issues suggest that current models are best suited for adjunctive educational and workflow roles rather than unsupervised clinical decision-making."[57] |
| **Theme 6: Adoption, Usability, and Implementation Barriers** | |
| Ease of use as a key factor in AI adoption | "The EG-ENT-R and EG-UPGY groups' emphasis on ease of use (PEoU score: 4.1) highlighted the advantages of the MEKAS system." [28] |
| Ease of use without model fine-tuning | "Finally, ENTAgents is ease of use. Since the RAG knowledge base is built by external sources and ENTAgents’s ability to self-correct derived from its own reflective critiques, generated through prompting alone, fine-tuning is not required within our framework. "[29] |
| High reproducibility across all topics | "Furthermore, the model demonstrated high reproducibility across all topics, and performed commendably without any significant differences between them." [23] |
| Higher perceived usefulness for research tasks than for studying | "To summarise, the item ”citation search” obtained satisfying responses, with 43% of residents answering a score of 4 and 14% a score of 5... Regarding the topic of ”content search,” 50% of residents answered with a score of 4 and 13% with a score of 5 ... Finally, none answered with a score of 5 for the usefulness of ChatGPT in studying..." [26] |
| Modest adoption among ENT trainees | "The analysis of our data reveals that the utilisation of ChatGPT remains modest among Italian ENT residents." "Despite its various functions and diverse range of applications, the adoption of ChatGPT for scientific purposes is still confined to a small number of ENT trainees." [26] |
| Primary use in research and academic study | "We identified two main areas of usage for ChatGPT by ENT trainees: research and study. We further divided the ”research” domain into three categories: citation searches, content searches, and language review." [26] |
| Equity risks in AI-enabled training | "Institutions with greater access to high-performing models and infrastructure may gain disproportionate educational advantages, potentially widening gaps in training quality across systems."[59] |
| Need for safe integration evidence | "Larger multicenter studies incorporating diverse question formats and updated artificial intelligence models are needed to define safe and effective integration strategies for artificial intelligence-assisted medical education."[60] |
| **Theme 7: Advanced AI Architectures and Future Educational Design** | |
| Accuracy and explainability of knowledge-based AI | "Conversely, the knowledge-based AI approach used in MEKAS offers accuracy and explainability, which is crucial for CCMK learning." [28] |
| Active learning through AI-generated assessment questions | "Considering the pedagogical principle that actively challenging exam questions enhances memory retention and learning outcomes compared to passive text review, AI-generated multiple-choice questions specifically designed for head and neck pathology study revision purposes can be generated as demonstrated in this paper. "[36] |
| AI-assisted instructional design and assessment development | "...to generate diverse case scenarios and differential diagnoses for teaching exercises.''; '' ...to create interactive learning modules where students/residents can query AI about morphologic features and diagnostic criteria."; "...may also develop assessment questions and rubrics for evaluating student understanding."; "...may use AI chatbots to create standardized patient scenarios for consistent teaching across multiple sessions." [36] |
| AI-assisted review and concept reinforcement | "Encourage students and residents to use AI chatbots for review, summarization, and clarification of concepts, particularly for board-style question practice or rapid recall. "[36] |
| Enhanced accuracy through retrieval-augmented generation (RAG) | "Utilizing agents and RAG can supplement the AI framework with up-to-date, highly specific, and detailed information, improving the accuracy and completeness of its responses. "[29] |
| Extensive search capabilities via agents and RAG | "The first one is extensive search capabilities. For example, ENTAgents draws information from both the RAG knowledge base and external sources through the use of agents. "[29] |
| Promotion of critical appraisal and analytical reasoning | "Instructors should guide learners to critique AI chatbot responses and understand reasoning errors, fostering analytical skills and diagnostic rigor." [36] |
| Self-correction through linguistic feedback | "The second is self-correction. This means that the reflection framework allows ENTAgents to evaluate their responses according to linguistic feedback." [29] |
| Self-reflective feedback for answer refinement | "These results show that ENTAgents has the capability to provide comprehensive answers with the aid of reflection critiques. The reflection critiques enable ENTAgents to comment on its answers and suggest what can be improved." [29] |
| Transforming medical education | "Recognizing the potential of AI chatbot technology in addressing the shortage of sleep medicine specialists, its application extends beyond patient care to reshaping the landscape of medical education, a sector poised for signifcant growth and transformation."[39] |
| Customizable synthetic cases | "It also has the ability to generate a large permutation of synthetic medical case studies and learning material that is customizable."[39] |
| Safe virtual-patient simulation | "LLMs also offer the potential for simulation-based learning, where residents and students can engage in virtual patient scenarios to enhance their diagnostic and therapeutic skills in a safe environment."[49] |
| Need for medical-image optimization | "To improve the accuracy of AI for image analysis, further studies on specialized prompts, the development of more advanced image-recognition technologies, and training focused on medical images are necessary."[61] |
| Specialty consultation potential | "In the future, LLM services may provide specific options for medical consultancy or accessible user training on specific ORL topics."[43] |
| Topic-specific user training | "In the future, LLM services may provide specific options for medical consultancy or accessible user training on specific ORL topics."[43] |
| Benefits of specialty fine-tuning | "In time, more specialized training and narrowing down to a thematic field could result in more accurate and concise responses."[43] |
| Domain-specific model outperforms comparators | "Generally, ChatENT demonstrated superior performance than both ChatGPT and human testers in MCQ questions, however, the margin is narrow.ChatENT is a pioneering entity in the realm of AI models, being the first LLM to specialize in domain‐specific knowledge in a specific surgical specialty. It exhibited superior performance to ChatGPT4.0 when tested on both multiple‐choice and short‐answer questions sourced from board examination materials."[44] |
| Need for expert-validated specialization | "Through targeted training tailored to different medical specialties and close validation by medical experts, GPT-4 models have the potential to transform into reliable, evidence-based tools that can be used in medical training, patient education, diagnosis guidance, and outcome prediction."[45] |
| Specialized instructions improve accuracy | "The higher accuracy demonstrated by Custom GPT-4 may be attributable to its instructions to specialize in Otolaryngology board-style questions, select one answer, and validate answers using the internet."[46] |
| Custom models support education | "Custom models may enhance utilization of ChatGPT in medical education."[46] |
| Enhanced education through customization | "This demonstrates custom models may further enhance utilization of ChatGPT in medical education."[46] |
| Interactive adaptive learning | "With the ability to interact with users, provide explanations, and adjust to user customizations, AI-based text models may continue to improve as tools for medical education."[46] |
| Need for domain refinement and interpretability | "Additionally future research should focus on refining and tailoring language models for specific domains, incorporating real-time learning mechanisms, and addressing the interpretability challenges associated with automated systems in complex decision-making processes within the medical field."[48] |
| AI-enabled knowledge-recall simulation | "AI could be integrated into exam simulations to quiz residents on basic knowledge recall, allowing residents to assess the strength of their fundamental knowledge."[54] |
| Customization improves specialty accuracy | "Achieving an accuracy of 74.6% on otolaryngology‐specific MCQs, the results underscore the potential benefits of customization in improving the relevance and accuracy of LLMs in specialized fields."[51] |
| Instruction tuning improves contextual relevance | "Customization through provided instructions allows for the fine‐tuning of language models to the specific language, terminology, and clinical nuances inherent in a particular medical field, thereby improving the model's ability to provide contextually appropriate—and presumably more accurate—responses."[51] |
| Supervised guideline-linked educational use | "From an implementation standpoint, these data support supervised, guideline-linked use in both education and decision support. For education, LLMs function well as case-discussion partners and formative-quizzing engines when prompts and expected outputs are standardized—an approach consistent with board-style studies and reports of domain-dependent performance"[58] |
| Reflective rather than passive engagement | "Our findings suggest that residents engaged with LLM output in a reflective rather than passive manner, particularly when discrepancies with their own plans were identified. Educational frameworks should therefore prioritize structured interaction models that promote metacognition rather than reliance."[59] |
| Need for medical-reasoning adaptation | "The observed plateau suggests that adaptation to medical reasoning patterns, rather than merely increasing scale, may be the critical next step."[57] |
| Hybrid reasoning support for complex cases | "Hybrid systems combining LLMs with structured knowledge bases or clinical decision trees might enhance performance on complex cases by providing explicit reasoning frameworks."[57] |
| Need for specialty fine-tuning | "These findings emphasize the need for domain-specific fine-tuning before LLMs can be reliably used in clinical education or decision support."[57] |
